# Design of metastable oxychalcogenide phases by topochemical (de)intercalation of sulfur in La_2_O_2_S_2_

**DOI:** 10.1038/s41467-021-23677-w

**Published:** 2021-06-14

**Authors:** Shunsuke Sasaki, Maria Teresa Caldes, Catherine Guillot-Deudon, Isabelle Braems, Gwladys Steciuk, Lukáš Palatinus, Eric Gautron, Gilles Frapper, Etienne Janod, Benoît Corraze, Stéphane Jobic, Laurent Cario

**Affiliations:** 1 Université de Nantes, CNRS, Institut des Matériaux Jean Rouxel, IMN, Nantes, F-44000 France; 2 Institute of Physics ASCR, v.v.i., Na Slovance 1999/2, Praha 8, 18221 Czechia; 3 Institut de Chimie des Milieux et Matériaux de Poitiers, 4 rue Michel Brunet, Poitiers cedex 09, 86073 France

**Keywords:** Chemical synthesis, Solid-state chemistry, Theoretical chemistry

## Abstract

Designing and synthesising new metastable compounds is a major challenge of today’s material science. While exploration of metastable oxides has seen decades-long advancement thanks to the topochemical deintercalation of oxygen as recently spotlighted with the discovery of nickelate superconductor, such unique synthetic pathway has not yet been found for chalcogenide compounds. Here we combine an original soft chemistry approach, structure prediction calculations and advanced electron microscopy techniques to demonstrate the topochemical deintercalation/reintercalation of sulfur in a layered oxychalcogenide leading to the design of novel metastable phases. We demonstrate that La_2_O_2_S_2_ may react with monovalent metals to produce sulfur-deintercalated metastable phases La_2_O_2_S_1.5_ and *oA*-La_2_O_2_S whose lamellar structures were predicted thanks to an evolutionary structure-prediction algorithm. This study paves the way to unexplored topochemistry of mobile chalcogen anions.

## Abstract (web summary)

Great progress has been made in topochemistry of mobile oxygen anions, but metastable compounds have not yet been achieved by deintercalation of sulfur anions. Here, the authors prepare metastable oxychalcogenide phases by taking advantage of redox-reactive sulfur dimers embedded in a layered oxysulfide.

## Introduction

Topochemistry enables a step-by-step control of chemical composition and atomic arrangements of materials by introducing, removing, or replacing specific atoms/molecules of host lattices without drastic modification of the overall crystal structure. It is one of the most versatile ways to design phases attainable only in mild synthesis conditions, i.e., at low temperature. Reactions aiming at the intercalation and the deintercalation of cationic species (mainly Li, Na, and other alkali metals) from an inorganic host have been extensively investigated since the sixties^1^. These topochemical reactions recently highlighted by the 2019 Nobel Prize in Chemistry led to the stabilization of numerous metastable phases^2–6^ and also gave rise to major applications in Li-ion batteries^7^. In 1999, Hayward and co-workers opened up a seminal topochemistry route based on the deintercalation of oxygen anions. Namely, they succeeded in extracting oxygen atoms of the perovskite-type oxide LaNiO_3_ at low temperature (*T* = 200 °C) using NaH as reducing reagent and oxygen getter to form the layered nickelate LaNiO_2_ with Ni^+^ cations in square planar coordination^8^. This synthetic pathway received much interest, as extraordinary changes in physical properties (e.g., magnetism, electrical conductivity) could be expected thanks to the stabilization of unusual oxidation states and chemical environment of cations while maintaining the low dimensionality of the inorganic framework^9–11^. In that respect the stunning discovery of the superconducting nickelate, Nd_0.8_Sr_0.2_NiO_2_, has recently spotlighted the benefit of this topochemical approach^12^. So far, such reactions were mainly limited to oxides and no topochemical deintercalation of sulfur at low temperature leading to metastable phases was reported.

To bring this situation to an end, we explore the intercalation/deintercalation of sulfur anions in lanthanum oxysulfides via an original soft chemistry route. We use the evolutionary structure prediction algorithm USPEX^13,14^, and 3D electron diffraction^15^ to confirm and characterize the topochemical deintercalation of sulfur and the formation of new metastable compounds. Return very recently, we demonstrated the formation of layered transition metal chalcogenides by insertion of transition metals in layered precursors containing chalcogenide dimers or trimers^16,17^. We took advantage of the ability of chalcogen oligomers to be easily reduced to form chalcogenide anions. In the case of sulfide dimers or trimers, the reductive cleavage (S_2_)^2-^ + 2e^-^ → 2 S^2-^ or (S_3_)^2-^ + 4e^-^ → 3 S^2-^ induced by contact with micrometric copper triggers the spontaneous intercalation of the metal into the precursor at low temperature. As a result, La_2_O_2_Cu_2_S_2_ and BaCu_4_S_3_ were obtained from the reaction of La_2_O_2_S_2_ or BaS_3_ with elemental copper at 250 °C. Our work also revealed that in the case of the reaction of BaS_3_ with Ni at 340 °C, sulfur could be extracted from the (S_3_)^2-^ containing precursor to form a (S_2_)^2-^ containing compound (e.g., BaS_2_) and a binary transition metal chalcogenide (e.g., NiS_x_)^17^. This observation suggests that the reduction of the oligomer by zero-valent metal can either lead to the intercalation of metal species or to the partial removal of chalcogen depending on the difference in free energies between the two antagonist reactions. It promoted us to explore more deeply the possible topochemical deintercalation of chalcogenides in layered precursors containing oligomers with various reducing reagents.

## Results and discussion

### Topochemical conversion from La_2_O_2_S_2_ to *oA*-La_2_O_2_S

**Fig. 1:**
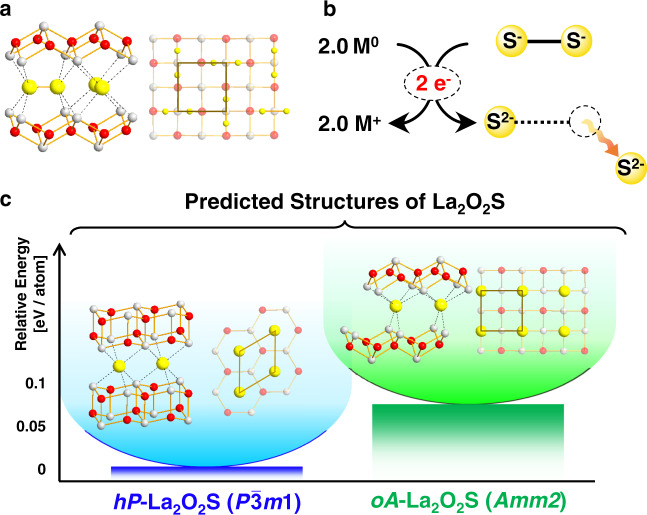
Topochemical reactivity of La_2_O_2_S_2_. **a** Structure of La_2_O_2_S_2_ reported by Ostorero et al. (SG: *Cmca*)^18^. Lanthanum, oxygen, and sulfur atoms are represented by white, red, and yellow balls, respectively. **b** Conceptual scheme of S–S bond cleavage under the donation of one electron per elemental metal M^0^ that triggers subsequently the deintercalation of half sulfur atom of the S_2_ dumbbell that possibly enables topochemical conversion of La_2_O_2_S_2_ into the new polymorph of La_2_O_2_S. **c** The two low-energy dynamically stable phases of La_2_O_2_S predicted by USPEX that are separated by 72 meV/atom.

La_2_O_2_S_2_ appears as the ideal precursor to test the possibility to extend such a concept of anionic topochemistry from oxides to chalcogenides (Fig. 1a). Its structure consists of fluorite-type ^2^/_∞_[La_2_O_2_]^2+^ infinite layers separated from each other by discrete (S_2_)^2-^ sulfur dimers aligned in parallel to these 2D blocks^18^. The deintercalation of one sulfur atom per dimer with metal should lead a priori to a La_2_O_2_S compound (Fig. 1b) whose structure should be inherited from the layered structure of the precursor La_2_O_2_S_2_ and that may come with potentially interesting optical properties as found in other oxysulfides^19,20^. To confirm this hypothesis, we started our investigation by scanning the low-energy structures of La_2_O_2_S compound using a designed crystal structure prediction (CSP) methodology detailed in Methods section. The combination of USPEX structure searching evolutionary algorithm with first-principles calculations makes it possible to locate two polymorphs, namely *hP* and *oA* crystal structures that are, respectively, stable and metastable (see Fig. 1c and S1–2 for details). Both phases are dynamically stable, justifying their respective location at global and local minima on the potential energy surface of La_2_O_2_S (see their computed phonon dispersion curves in Figs. S3–4, as well as DFT details in SI). The most stable candidate exhibits a hexagonal layered structure with ^2^/_∞_[La_2_O_2_] fluorite-type (111) slab alternating with sulfur atoms in octahedral environment of lanthanum (*P*$\bar{3}$*m*1 space group). Note that, this is the exact structure of the La_2_O_2_S compound already reported in the literature^21^, and commonly prepared at high temperature (800–1200 °C). This is interesting as it gives a direct validation of the modeling approach. In the following, this structure will be denoted *hP*−La_2_O_2_S according to the Pearson notation (*h* for hexagonal and *P* for primitive cell). The second low-energy phase lies at 72 meV/atom above ground-state *hP*-La_2_O_2_S (GGA PBE level of theory, see SI). Its structure is fully reminiscent to the one of the La_2_O_2_S_2_ precursor: it is built upon the stacking of ^2^/_∞_[La_2_O_2_] fluorite-type (001) slabs alternating with sulfur atoms in prismatic environments. In the same way with *hP*-La_2_O_2_S, this metastable polymorph with orthorhombic *Amm*2 space group is named hereafter *oA*-La_2_O_2_S. The thermal and kinetic stability of these two structures were further confirmed by ab initio molecular dynamics (AIMD) simulation in which both *hP*- and *oA*−La_2_O_2_S retained their main structural framework after 10 ps at temperatures up to 600 K (Fig. S5–S6). Consequently, the theoretical calculations clearly anticipate the possible existence of metastable *oA*−La_2_O_2_S besides the already known phase *hP*-La_2_O_2_S.

**Table 1:** Summary of crystallographic parameters of La_2_O_2_S_*x*_ series (1 ≤ *x* ≤ 2.0).

Compounds	Source	Space group	*a* (Å)	*b* (Å)	*c* (Å)	S–S distance (Å)
La_2_O_2_S_2_	Ostorero et al^18^.	*Cmca*	13.215(2)	5.943(1)	5.938(1)	2.103
*hP*-La_2_O_2_S	Morosin et al^21^.	*P*$\bar{3}$*m*1	4.049(1)	-	6.939(2)	4.049
*oA*-La_2_O_2_S	This study	*Amm*2	4.148(9)	3.975(1)	12.728(0)	3.975(1)
*oA*-La_2_O_2_S_1.5_	This study	*Amm*2	8.4759(5)	4.0307(1)	12.853(0)	2.30(6)

All of the indicated values were obtained from refinement of a respective XRD pattern.

**Fig. 2:**
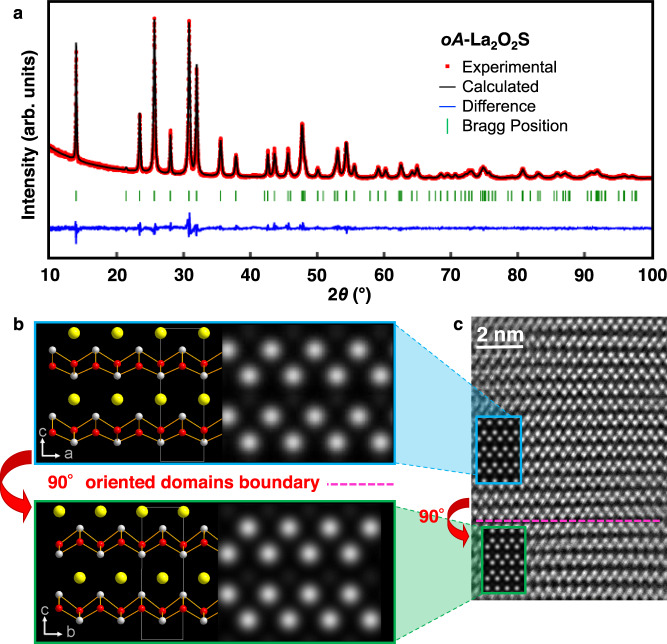
Structure characterization of *oA*-La_2_O_2_S. **a** Experimental XRD pattern of *oA*-La_2_O_2_S obtained by the reaction of La_2_O_2_S_2_ with Rb^0^ metal at 350 °C. The Rietveld refinement was conducted using as input the structure predicted by USPEX. **b** Simulated HAADF-STEM images viewed along [010] and [100] directions obtained using the refined structure of *oA*-La_2_O_2_S. In the projected structure lanthanum, oxygen, and sulfur atoms are represented by white, red, and yellow balls, respectively. **c** The experimental HAADF-STEM image of *oA*-La_2_O_2_S superimposed by each of the simulated images. Only La atoms were observed as bright spheres (ADF signal is proportional to the *n*th power of the atomic number, with *n*~2).

The topochemical deintercalation of sulfur in the layered precursor La_2_O_2_S_2_ was subsequently attempted by reaction with alkali metal Rb^0^ at low-temperature in evacuated and sealed pyrex tubes. Once excess Rb (and its salts) was washed out by dry ethanol (see synthetic procedure in Methods section), the powder X-ray diffraction (XRD) patterns were collected on products synthesized at 200 °C and 350 °C. They are very similar and could not be indexed with any known phases (Fig. 2a). The energy dispersive X-ray (EDX) spectra of the bulk product powder clearly revealed the absence of rubidium and a molar ratio La / O / S of 2.0(3) / 2.0 (4) / 0.98(11) (see Fig. S7). These results indicate the formation of a La_2_O_2_S phase without incorporation of Rb in the structure. The *hP*-La_2_O_2_S XRD peaks were not detected at all in the X-ray pattern but the existence of the polymorph *oA*-La_2_O_2_S predicted by USPEX could be readily established via Rietveld refinement (Fig. 2a) with goodness of fit *χ*^2^ = 1.33 and Bragg reliability factor *R*(obs) = 1.68% (see Table S3 for details). Scanning Transmission Electron Microscopy (STEM) also support the conclusion that the newly synthesized phase is *oA*-La_2_O_2_S. The stacking of ^2^/_∞_[La_2_O_2_] infinite sheets with the fluorite-type (001) slab structure is clearly visible on the High Angle Annular Dark Field (HAADF) STEM image (Fig. 2b, c). In contrast, the fluorite-type (111) slabs characteristic of the stable polymorph *hP*-La_2_O_2_S (Fig. S8) could not be found in the experiment STEM image. The EDX spectrum of a nanosized single crystal indicated, similarly to the EDX analysis of the bulk powder, a La / S ratio of 2.0(3) / 1.0(2) consistent with the composition La_2_O_2_S (see Fig. S9). The structural arrangement of the new *oA*-La_2_O_2_S compound is directly inherited from La_2_O_2_S_2_. This observation definitely supports the topochemical nature of the deintercalation process. Indeed, the sulfur deintercalation process does not modify at all the integrity of the ^2^/_∞_[La_2_O_2_] slab but entails a shift of every second ^2^/_∞_[La_2_O_2_] layer along the ½(*b* + *c*) direction of the La_2_O_2_S_2_ structure (SG: *Cmca*). Raman spectroscopy confirmed the complete loss of the sulfur dimers along the topochemical reduction. Namely, the band associated to the S–S stretching modes located at 487 and 498 cm^−1^ in La_2_O_2_S_2_ have totally disappeared after the deintercalation of one sulfur from La_2_O_2_S_2_ (Fig. S10), confirming the conclusion made from the XRD pattern that the reaction of La_2_O_2_S_2_ towards *oA*-La_2_O_2_S was complete. Finally, the diffuse-reflectance spectra also support the cleavage of (S_2_)^2-^ dimers (Fig. S11). The absorption thresholds move from 2.56 eV in La_2_O_2_S_2_, a value characteristic of a π*–σ* electronic transition of sulfur pairs^22,23^, to 3.88 eV in *oA*-La_2_O_2_S, a value significantly lower than the one observed in the *hP*-La_2_O_2_S (4.13 eV). Our DFT calculations with the HSE06 functional fully confirmed the expected increase of the bandgap going from *oA*-La_2_O_2_S to *hP*-La_2_O_2_S (calculated gaps of 4.1 and 4.3 eV, respectively). Based on previous experimental and theoretical studies on *hP*-La_2_O_2_S^24,25^, their optical bandgaps can be associated to a S-3p → La-6s/La-5d transition. Therefore, the difference of the bandgap values between the metastable and stable La_2_O_2_S varieties is likely ascribed to changes in the local environment of sulfur and lanthanum atoms.

As a whole, we can conclude that during the reaction between La_2_O_2_S_2_ and elemental rubidium, the alkali metal activates a redox reaction with (S_2_)^2-^ dimers that triggers the fracture of the S–S bonds. However, contrary to Cu^0^ metals that intercalate into the La_2_O_2_S_2_ host lattice^16^, Rb^0^ led to the topochemical deintercalation of sulfur to afford the unprecedented *oA*-La_2_O_2_S metastable phase. Preliminary calculations suggest that the intercalation of metal versus the deintercalation of sulfur is dictated by thermodynamic considerations and that the choice of reducing agents is the decisive factor for the outcome of the reaction. Even though, complete theoretical investigations are needed to ascertain this assertion, some experimental work further highlight the decisive role of the reducing power. Indeed, no reaction occurred when La_2_O_2_S_2_ was treated at 200–300 °C under reducing atmosphere, i.e., 5% H_2_/Ar flow (Fig. S12). The reduction was activated at 350 °C, but it led to the thermodynamically stable *hP*-La_2_O_2_S, where the original fluorite (001) slab was deformed into the fluorite (111) slab. This result highlights the contrast between the common reducing agent such as H_2_ atmosphere and the more powerful reducing agent Rb^0^, which favored, even at the same reaction temperature (i.e., 350 °C), topochemical reduction to *oA*-La_2_O_2_S.

### Partial (de)intercalation of sulfur

**Fig. 3:**
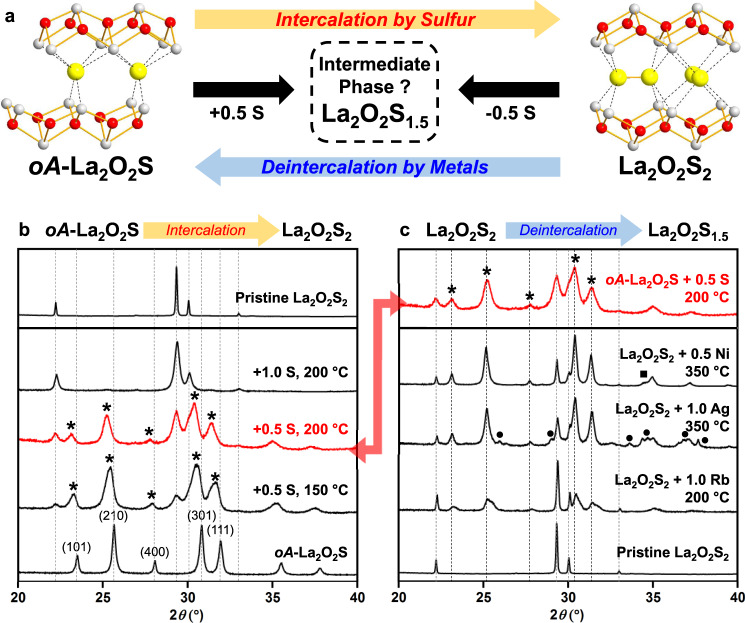
Incomplete intercalation and deintercalation of sulfur. **a** Schematic representation of incomplete intercalation of sulfur into *oA*-La_2_O_2_S and deintercalation of sulfur from La_2_O_2_S_2_ that leads to an intermediate compound La_2_O_2_S_1.5_. **b** Intercalation experiments of sulfur into *oA*-La_2_O_2_S. Experimental XRD patterns of pure *oA*-La_2_O_2_S and of the products of its mixture with sulfur (0.5 or 1 equiv. of S) after thermal treatments at 150 or 200 °C. The new XRD peaks emerging after the thermal treatment with 0.5 S are marked by *. **c** Deintercalation of sulfur from La_2_O_2_S_2_. Experimental XRD patterns of La_2_O_2_S_2_ and of the product of its mixture with Rb^0^, Ag^0^, and Ni^0^ after the thermal treatments at 200 or 350 °C. The XRD peaks of minor by-products are marked as follows: ● = Ag_2_S^37^, ■ = *α*-NiS^38^.

At this stage, we might naturally wonder whether the topotactic deintercalation of La_2_O_2_S_2_ is reversible or not at low temperature. To test this possibility, a portion of *oA*-La_2_O_2_S was mixed with one molar equivalent of sulfur and heated at 200 °C (Fig. 3a) and the product was analyzed by XRD (Fig. 3b). The original La_2_O_2_S_2_ material could be fully recovered with no other reaction product, confirming the full reversibility of the temperature-assisted intercalation/deintercalation processes based on the formation/rupture of sulfur dimers within the La_2_O_2_S/La_2_O_2_S_2_ layered oxychalcogenides. To gain more insight into the reaction mechanism of the sulfur intercalation, we also tested the reactivity of *oA*-La_2_O_2_S with only half molar equivalent of sulfur at low temperature to reach the target La_2_O_2_S_1.5_ compound, i.e., a mid-composition between La_2_O_2_S_2_ and La_2_O_2_S. The XRD pattern of the product obtained at 200 °C (Fig. 3b) revealed the conversion of *oA*-La_2_O_2_S into an unknown intermediate phase along with a small amount of La_2_O_2_S_2_. The XRD pattern of this intermediate phase was similar to the one of *oA*-La_2_O_2_S but shifted to lower diffraction angles, suggesting the existence of an intercalated *oA*-La_2_O_2_S_*x*_ phase (1 < *x* < 2.0). The same XRD pattern was observed in the attempt to deintercalate 0.5 S from La_2_O_2_S_2_ using 1.0 equiv. of Rb^0^, 1.0 equiv. of Ag, and 0.5 equiv. of Ni^0^ (Fig. 3c). These diffraction patterns could be refined with the same space group as *oA*-La_2_O_2_S (*Amm*2) and cell parameters of ~8.5 Å, ~4.0 Å, and ~12.9 Å without any superstructure peak. This clearly proved the existence of an intermediate phase with a strong reminiscence of the *oA*-La_2_O_2_S structure. One of the reasonable assumptions is that this new phase replaced one half of monoatomic S^2-^ with dimeric (S_2_)^2-^ anions retaining the main structural framework of *oA*-La_2_O_2_S. This partial dimerization should lead to the expected La_2_O_2_S_1.5_ composition. To confirm this hypothesis, we analyzed the elemental composition of the nanocrystal (≤100 nm) found in the sample after the thermal treatment of *oA*-La_2_O_2_S + 0.5 S. Its EDX spectrum acquired on STEM indicated the La / O / S ratio of 2.0(3) / 2.0(3) / 1.5(3), supporting the hypothetical composition La_2_O_2_S_1.5_ (see Fig. S13). Furthermore, both intercalation of 0.5 S and deintercalation of 0.5 S using metal species gave similar Raman spectra that featured a single intense peak at 413–417 cm^−1^ while Raman peaks from *oA*-La_2_O_2_S and La_2_O_2_S_2_ were absent (Fig. S14–S15). Since an intense peak around 400–500 cm^−1^ is characteristic of S–S stretching mode^26^, these Raman spectra support the formation of La_2_O_2_S_1.5_ through the partial cleavage of S–S bonds.

**Fig. 4:**
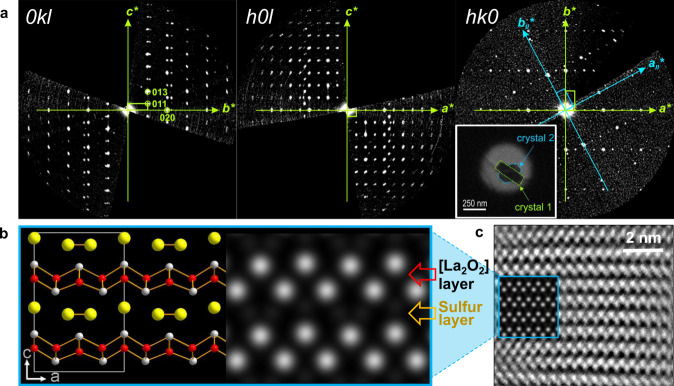
Structure characterization of *oA*-La_2_O_2_S_1.5_. **a** Projection of the reciprocal space obtained from PEDT data following the indexing of the first crystal (green). The second visible lattice (blue) corresponds to a second adjacent crystal and was measured in the same data set. **b** The structure of *oA*-La_2_O_2_S_1.5_ solved and refined from PEDT data set. The right side of the panel represents the simulated HAADF-STEM image along [010] on the basis of the *oA*-La_2_O_2_S_1.5_ structure model. **c** The experimental HAADF-STEM image acquired in a well-organized domain *oA*-La_2_O_2_S_1.5_ superimposed with the simulated one.

To solve the crystal structure of this novel phase, we analyzed it by precession electron diffraction tomography (PEDT) (Fig. 4a, see Fig. S16 and Table. S4 for details)^27,28^. This emerging technique allows the solution of complex structures using single nanocrystals. PEDT data were collected on several nanocrystals of the novel phase. All data sets were analyzed using the computer programs PETS2.0^29^, Superflip^30^, and Jana2006^31^. Figure 4a shows the reconstruction of the reciprocal lattice planes *hk*0, *h0l*, and 0*kl* which are consistent with an orthorhombic unit cell *a* = 8.348 Å, *b* = 3.961 Å, and *c* = 12.645 Å (*V* = 418.1 Å^3^) and a non-centrosymmetric space group *Amm*2 (see SI). The structure was subsequently solved and refined using the program Jana2006 on the basis of electron diffraction data using the dynamical theory of diffraction^32,33^. The structure analysis converged with electron Bragg reliability factor *R*(obs) = 10.1%, revealing the layered structure displayed in Fig. 4b with the composition La_2_O_2_S_1.5_. This structure consists of ^2^/_∞_[La_2_O_2_] fluorite-type (001) infinite slab alternating with sulfur layers containing one third and two thirds of sulfur atoms in the S^2-^ and (S_2_)^2-^ anionic forms, respectively. Using this *oA*-La_2_O_2_S_1.5_ structure model, both powder XRD patterns from sulfur intercalation and deintercalation, i.e., from *oA*-La_2_O_2_S + 0.5 S and La_2_O_2_S_2_ + 0.5 Ni reaction mixtures (Fig. 3), were successfully refined (Fig. S17 and Tables S5–S6). Large strain parameters had to be considered to reach satisfactory fitting. This can be interpreted as the signature of a stacking disorder occurring, as expected, during the intercalation or deintercalation processes in relation with the 2D structure of the host lattice and possible co-existence of different stages of (de)intercalation. The structure analysis was based on data collected on the most ordered crystals. However, in most of the electron diffraction data, diffuse scattering features along the [001] axis signified the presence of stacking faults in the crystal. Fig. 4c shows the high-resolution image obtained on a well-organized domain of *oA*-La_2_O_2_S_1.5_. The experimental contrast in the HAADF-STEM image (Fig. 4c) asserts the stacking of ^2^/_∞_[La_2_O_2_] fluorite-type (001) infinite slabs. A similar structure was predicted independently by the evolutionary algorithm USPEX for the composition La_2_O_2_S_1.5_. Indeed, the structure prophesied to be the most stable accorded well with the experimental structure obtained by the PEDT analysis (Fig. S18). The 2^nd^ and 3^rd^ most stable structures displayed intergrowth structures made of, respectively, corrugated or hexagonal (fluorite-type (111)) 2D [La_2_O_2_] slabs alternating with (quasi-)2D arrays of sulfur dimers/atoms. However, none of them could be found in our experiments.

**Fig. 5:**
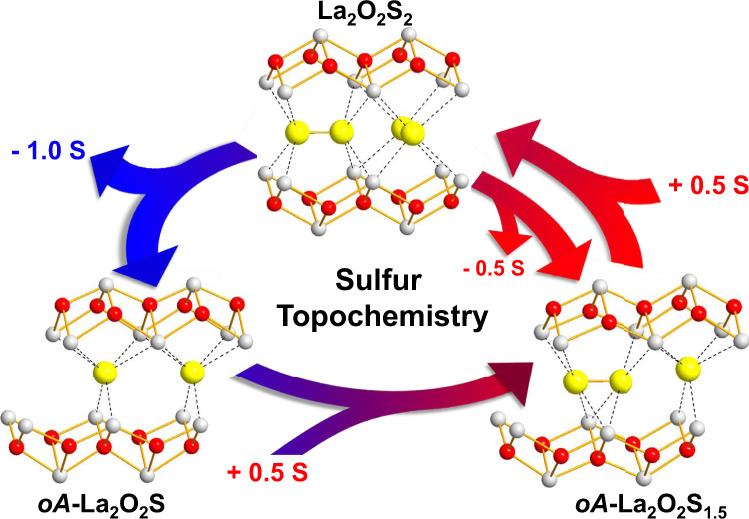
Overview of the rich low-temperature sulfur topochemistry in the La–O–S system. The topochemical intercalation and deintercalation of sulfur in the oxychalcogenide compound La_2_O_2_S_2_ lead to the formation of two new metastable compounds.

To conclude, this work demonstrates the deintercalation and reintercalation of sulfur in a layered oxychalcogenide compound using an original topochemical approach. Alkali or transition metals may be used as reducing agent to trigger the reduction of the chalcogenide oligomers and breaking of the chalcogen–chalcogen bond. In the case of La_2_O_2_S_2_, the low-temperature deintercalation of sulfur atoms proceeds in two steps to form two new metastable phases La_2_O_2_S_1.5_ and *oA*-La_2_O_2_S that both retain the layered feature of the precursor (Table 1). This is a fully reversible topotactic process as the sulfur atoms may be re-intercalated at low temperature to form back the precursor La_2_O_2_S_2_ (illustrated schematically in Fig. 5). This work gives therefore a glimpse of what could be the richness of the topochemistry of chalcogenide compounds and may open up an avenue to forthcoming batteries built upon mobile sulfur ions as well as to promising electronic or optical materials^19,23–25^.

## Methods

### Topochemical conversion from La_2_O_2_S_2_ into *oA*-La_2_O_2_S

The initial precursor La_2_O_2_S_2_ was synthesized following the procedure described in our previous report^16^. La_2_O_2_S_2_ and Rb (Aldrich, 98 + %) were weighed in a 1: 2 molar ratio and introduced into a silica tube whose bottom was protected by a carbon coating (cracking of acetone in the flame). All these preparations were done under argon atmosphere, then the silica tube was evacuated (~10^−3^ torr) and sealed. The mixture was heated to 200 °C at a rate of 20 °C h^−1^ and held at that temperature for 2 h. Finally, the sealed tube was gradually cooled to room temperature. The obtained powder turns to be pale greyish-blue. The silica tube was opened under argon atmosphere and the content was washed with ethanol, which rendered the powder colorless. The colorless precipitate was contaminated by tiny flakes of carbon, which were removed by repetitive decantation with mechanical agitation. The precipitate was then washed with ethanol, water, and acetone, followed by dryness in vacuo to obtain the pure colorless powder of *oA*-La_2_O_2_S. The product was stable under ambient atmosphere. The same reaction performed at 350 °C gave identical results, i.e., pure *oA*-La_2_O_2_S without any trace of *hP*-La_2_O_2_S.

### Intercalation of sulfur anions into *oA*-La_2_O_2_S

The colorless powder of *oA*-La_2_O_2_S (ca. 200 mg) was combined with S flakes (Aldrich, 99.99 + %) in *oA*-La_2_O_2_S: S = 1: 0.5 molar ratio and ground in an agate mortar under argon atmosphere. Then the mixture was pelletized and sealed in an evacuated (~10^−3^ torr) silica tube. The sealed mixture was heated to 150 or 200 °C at a rate of 100 °C h^−1^ and held at the same temperature for 4 h (*T* = 200 °C) and 48 h (*T* = 150 °C, see results in Fig. 3b), respectively, followed by gradual cooling in a furnace to obtain a pale yellow pellet. To complete the intercalation, the obtained pellet was ground with additional 0.5 equiv. of S under argon atmosphere. The mixture was again subject to the thermal treatment at 200 °C in the evacuated silica tube. After 160 h of annealing, the mixture was fully converted into the pale yellow pellet of the pure La_2_O_2_S_2_.

### Partial deintercalation of sulfur from La_2_O_2_S_2_ to get *oA*-La_2_O_2_S_1.5_

To prepare *oA*-La_2_O_2_S_1.5_, either 0.5 equiv. of Ni (Aldrich, <100 nm, 99%) or 1.0 equiv. of Ag (Aldrich, 2–3.5 μm, ≥99.9%) was added to 1.0 equiv. of La_2_O_2_S_2_ (ca. 150–250 mg). The mixture was ground together under argon atmosphere until the powder became greyish and sticky. Then the powdered sample was pelletized, sealed in an evacuated (~10^−3^ torr) silica tube, and heated to 350 °C at a rate of 300 °C h^−1^.The plateau temperature was maintained for 2 or 4 h before gradual cooling at RT for Ni and Ag, respectively (see Fig. 3c for XRD). Repeated thermal treatments did not lead to further consumption of La_2_O_2_S_2_ even when excess Ag was added (i.e., using 1.1 equiv. of Ag). In the same way, when using Ni prolonged and repeated thermal treatments did not improve the yield of *oA*-La_2_O_2_S_1.5_.

### Characterizations

Powder X-ray diffraction (XRD) patterns were recorded at room temperature on a Bruker D8 Advance Diffractometer (Bragg-Brentano geometry, *θ*−2*θ*), which employs Cu K_*α*1_ radiation (*λ* = 1.540598 Å) produced through Ge (111) monochromator and a LynxEye detector. Rietveld refinements of these XRD patterns were performed with Jana2006 package^31^ using the fundamental parameter approach^34^. XRD peaks from the known phases were fitted on the basis of the previously reported structure models (see Table S3 and S5–6 for the details). Anisotropic microstrain was considered by refining anisotropic peak broadening employing Stephen’s tensor method^35^. The chemical composition of *oA*-La_2_O_2_S was inspected by energy-dispersive X-ray (EDX) spectroscopy on a JEOL 5800LV scanning electron microscope (SEM) operating at 20 keV. The flat-polished specimens were prepared by impregnation of powder samples with the epoxy resin and subsequent grinding with ethanol/diamond grit suspensions. The detailed procedures of Raman spectroscopy and diffuse-reflectance spectroscopy are described in the supporting information.

### Scanning Transmission Electron Microscopy (STEM) imaging

Samples were prepared by dispersing the powder in ethanol and depositing the solution obtained on a holey-carbon-coated copper grid. Z-contrast imaging was performed on a Cs-probe corrected STEM Themis Z G3 (Thermo Fisher Schientific) equipped with a High Angle annular Dark Field (HAADF) detector (Fischione) operating at 80 or 300 kV accelerating voltage, with a 21.4 mrad convergence angle and 63–200 mrad collection angle. It is equipped with the 4-SDD detectors Super-X system allowing EDS analysis. Simulated images were obtained using the Dr Probe program^36^.

### Precession Electron Diffraction Tomography (PEDT) of *oA*-La_2_O_2_S_1.5_

The black powder from the reaction of La_2_O_2_S_2_ with Ni (*vide supra*) was crushed in ethanol. A drop of the suspension was deposited and dried on a gold grid with a thin film of holey amorphous carbon. 3D electron diffraction data (3D ED)^15^ were collected using the precession electron diffraction tomography (PEDT) technic on a Philips CM120 electron transmission microscope (TEM) (*V*_acc_ = 120 kV, LaB_6_) with the precession device Nanomegas Digistar and a side-mounted CCD camera Olympus Veleta with 14 bit dynamic range. PEDT data sets were collected at the ambient temperature on several crystals using a 1-degree precession angle and a 1-degree tilt step (see Fig. S16 and Table. S4). 3D ED data were analyzed using the computer programs PETS2.0^29^, Superflip^30^, and Jana2006^31^. The structure analysis was performed from one data set that included the diffraction signal from two crystals (Fig. S16). For each data set, two *hkl*-type files are obtained: one for structure solution and kinematical refinement, and another file suitable for the dynamical refinement, where each ED frame is considered independent^32,33^. The refinement procedure using the dynamical theory of diffraction (called “dynamical refinement”) implemented in JANA2006 was used . For the analysis, the data coming from the two adjacent crystals were combined to increase the data coverage and the statistic of the refinement. The data indicate an orthorhombic unit cell *a* = 8.348 Å, *b* = 3.961 Å, and *c* = 12.645 Å (*V* = 418.1 Å^3^) and a non-centrosymmetric space group *Amm*2 (*k* + *l* = 2n on *hkl*). The initial structure can be described with 8 atoms forming an alternation of infinite fluorite-type layers [La_2_O_2_] and sulfuric layers stacked along *c*-axis (Fig. S16e). The model was validated by the dynamical refinement following the procedure suggested in Palatinus et al.^32,33^. The refinement leads to satisfying values with *R(obs)/wR(obs)* = 10.12%/11.42% for 3523/4246 observed/all reflections. The refinement and crystallographic parameters are presented in Table S4.

### Structure predictions by the evolutionary algorithm

USPEX (Universal Structure Predictor: Evolutionary Xtallography) works on the EA (evolutionary algorithm) to perform a global search of crystal structures. USPEX is developed by the A.R. Oganov laboratory since 2004 and its detail is described in the articles from his group^13,14^. In this work, this EA code was interfaced with VASP (Vienna Ab initio Simulation Package) for DFT structure relaxation (shape, volume, atomic positions are optimized by VASP). The EA development allows the prediction of stable and metastable structures knowing only the chemical composition, i.e., 2 La and 2 O and 1 S here, to search 3D La_2_O_2_S bulk phases. An initial population of 80 randomly created structures was considered as the first generation. Then the structures considered as worst were discarded. A structure was considered bad according to its fitness, that was the computed free enthalpy derived from ab initio total energy calculations (VASP). The remaining structures form the parent structures participated in producing the next generation. A new candidate structure was produced from parent structures using one of four operators: (i) heredity (50%), (ii) permutation (10%), (iii) lattice mutations (10%), and (iv) soft mutation (10%). 80% of the new candidates were generated from evolutionary operators, while 20% were produced randomly. We verified that our results were not sensitive to these parameters (initial population size, ratio between the four operators), by performing multiple runs with them varying from 10% around the nominal value indicated here. The USPEX search was terminated when the global structure minimum was found in the last 10 generations. Each USPEX job was run at least twice to ensure the convergence to a global minimum. As mentioned above, structure relaxations and energy calculations were done by the external code VASP (5 INCAR files, 5 steps per phase). The total number of atoms in the primitive cell is up to 40.

## Supplementary information

Supplementary Information

## Data Availability

All data are available within the Article and Supplementary Files, or available from the corresponding authors on reasonable request.
