# Supplementary material for: Design of metastable oxychalcogenide phases by topochemical (de)intercalation of sulfur in La2O2S2
Source: Nat Commun. 2021 Jun 14;12:3605. doi: 10.1038/s41467-021-23677-w (PMC8203606; doi:10.1038/s41467-021-23677-w)
Supplement: Supplementary file 1 — Supplementary Information [file 41467_2021_23677_MOESM1_ESM.pdf]

Supplementary Information

Design of Metastable Oxychalcogenide Phases by Topochemical (de)intercalation of Sulfur in $La_2O_2S_2$

Experimental procedures

1. Characterization methods: Raman spectroscopy

Raman spectra were recorded in a backscattering geometry with a 514 nm argon laser using Renishaw inVia Raman spectrometer. The laser power was adjusted to 0.15 mW on the sample but was limited to 0.015 mW when the Raman signals saturated (the laser power used for each acquisition was noted in Fig. S10 and S14-S15). For all measurements, at least two different points on powder samples are analyzed to ensure homogeneity of samples.

2. Characterization methods: Diffuse-reflectance spectroscopy

The diffuse-reflectance spectra were recorded on a Varian Cary 5G spectrometer equipped with a 60 mm diameter integrating sphere and computer control using the Cary WinUV software. Diffuse reflectivity was collected from 250 to 800 nm (i.e. 1.55 – 4.96 eV) as Kubelka-Munk function $K / S = (1 - R)^2 / (2R)$, where sample reflectance R was calibrated using Halon powder (Varian) as the reference of 100% reflectivity.[1] The obtained Kubelka-Munk function K / S was directly employed for estimation of optical band gap E_g without converting them into the Tauc plots.[2-3]

3. Computational methodology

Density functional theory (DFT) calculation for structure optimization

First-principles calculations are performed using the projected-augmented-wave (PAW) method as implemented in VASP (version 5.4.4). In USPEX calculation, exchange-correlation energy is treated using Perdew-Burke-Ernzerhof (PBE) within the generalized gradient approximation (GGA PAW potentials for lanthane, oxygen and sulfide atoms have a radius of 2.8 au for La ([Kr] core), 1.52 for O ([He] core) and 1.9 au for S ([Ne] core), respectively. To optimize each crystalline structure, 5 successive steps of increasing convergence accuracy are usually required (i.e. 5 INCAR files). The parameters and criteria associated with VASP calculations then correspond to the last (5th) step of the highest accuracy. A kinetic cutoff energy of 550 eV is used for the wavefunction expansion with a Monkhorst-Pack *k*-mesh grid with 5 subdivisions along the

reciprocal lattice vectors. All structures are optimized until the net forces on atoms are below 1 meV/Å, and all forces on atoms are converged to less than 0.005 eV/Å.

The series of structures predicted by USPEX algorithm fell into three structure types after the relaxation at the PBE level of theory but one of these three structure with monoclinic $P2_1/m$ space group was found to be dynamically unstable with negative phonon frequency (*vide infra* for calculation procedure). Therefore, this monoclinic structure was excluded from further considerations. Other two dynamically stable structures were subject to further optimization with the strongly constrained and appropriately normed (SCAN) meta-GGA functional[4] combined with the revised Vydrov–van Voorhis nonlocal correlation (rVV10).[5-6] SCAN+rVV10 functional allows to treat the weak dispersion forces of layered structures and therefore it is suitable to estimate the interlayer spacing as well as intra-layer lattice parameters of La_2O_2S whose predicted structures are lamellar. The optimization at SCAN+rVV10 level of theory increased the difference in the relative enthalpy between each structure type (Table S2). On the other hand, their structural parameters were hardly varied by the inclusion of van der Waals effects. Thus, the structures given at the PBE level of theory were used for other calculations (*vide infra*) and the comparison with our experimental data.

For better estimation of the optical band gap (E_g) of La_2O_2S phases, the single-point energy calculation using the optimized structure was performed at the Heyd−Scuseria−Ernzerhof (HSE06) hybrid functional level of theory.

Phonon calculations

In this work, first principles phonon calculations using the Density functional perturbation theory (DFPT) at a quasi-harmonic level are done using the open source package PHONOPY.[7] Supercell structures with or without displacements are created from a reference La_2O_2S unit cell considering all possible crystal symmetry operations. To avoid unphysical imaginary frequencies, a 2x2x2 or a 3x3x2 supercell are used. Force constants are calculated using the optimized structure (VASP). The Brillouin zone and the associated k-path of each structure are computed using the Bilbao crystallographic server tools.[8]

Ab Initio molecular dynamics (AIMD)

Ab initio molecular dynamics (AIMD) with canonical ensemble using the Nosé heat bath scheme were performed to evaluate the thermal stability of specific phases up to 1500 K for 10 ps, for a timestep of 1 fs. The simulations based on DFT are also carried out using VASP code to examine the candidate phases (PBE level of theory). In such AIMD simulations, the Brillouin zone

integration is restricted to the Γ point of the supercell, due to a high calculation cost. A 2x2x2 supercell is used (up to **160** atoms par repeat unit). A metastable crystalline structure is definitively termed viable if no structural transformations are observed in the La-S-O network, i.e., an absence of La-O, and La-S bond breaking after a long simulation (10 ps), indicating the existence of a substantial kinetic barrier that facilitates formation and trapping of viable (meta)stable structures.

Computational results

1. Crystal and energy parameters of investigated stable and metastable structures

Table S1. Structural parameters and space groups of the two structure types predicted for the fixed composition La_2O_2S (distances in Å, angle in °).

Phase name	*Z*	Space Group	Lattice parameters	Atomic coordinates (fractional)	Volumes (Å^3/atom)
hp	1	$P\bar{3}m1$ (164)	$a = b$ =4.120<br>c = 6.615<br>$\alpha = \beta$ = 90.0<br>γ =120.0	La1: (1/3, 2/3, 0.27720)<br>O1 : (1/3, 2/3, -0.36661)<br>S1 : (0, 0, 0)	459
oA	2	*Amm*2 (38)	a = 4.142<br>b = 4.012<br>c = 12.706<br>$\alpha = \beta = \gamma$ = 90.0	La1 : (0.0, 0.0, -0.16352)<br>La2 : (0.5, 0.0, -0.47031)<br>O1 : (0.5, 0.0, -0.06718)<br>O2 : (0.0, 0.0, 0.44459)<br>S1 : (0.5, 0.0, 0.21191)	230

Table S2. Computed relative energy *hP*- and *oA*-La_2O_2S phases at the PBE and SCAN+rVV10 levels of theory.

Structure	Δ*E* in PBE (eV/atom)	Δ*E* in SCAN+rVV10 (eV/atom)
hp-La_2O_2S	+0.00	+0.00
oA-La_2O_2S	+0.072	+0.396

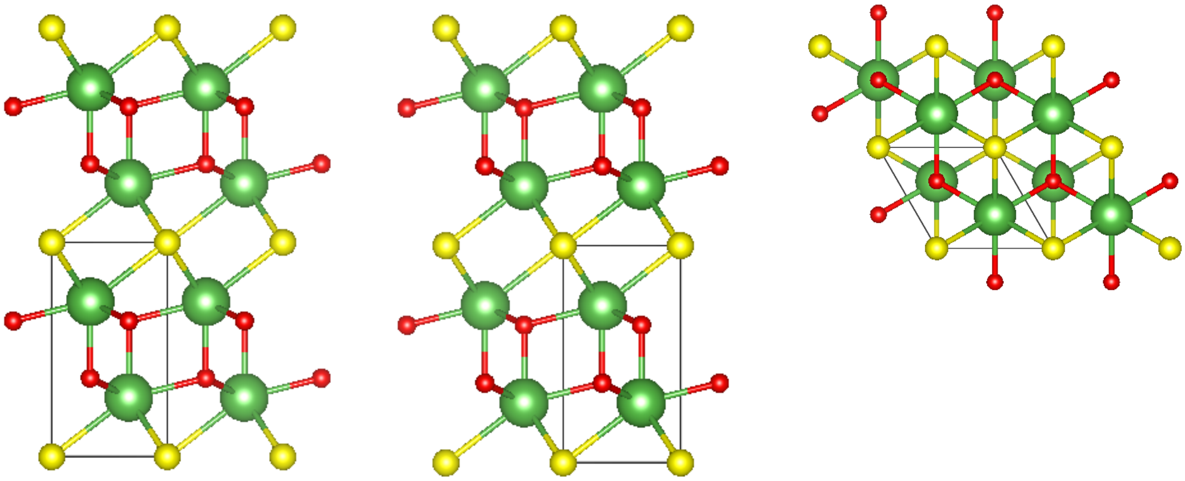

Figure S1. The lowest-energy structure *hP*-La_2O_2S predicted by the evolutionary algorithm USPEX (See Table S1 for its structural parameters). These images are viewed along (left) *a*-axis, (middle) *b*-axis, and (right) *c*-axis. Lanthanum, oxygen and sulfur atoms are represented by the green, red and yellow spheres respectively. The structure can be classified into the aphthitalite type, where the $(La_2O_2)^{2+}$ monolayer possesses hexagonal symmetry and sulfur atoms lie on the same plane without corrugation.

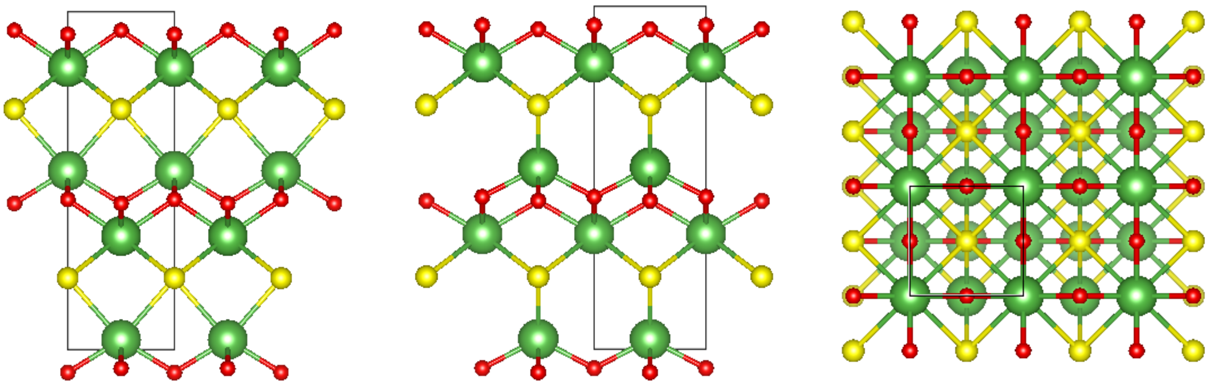

Figure S2. The 2nd lowest-energy structure of *oA*-La_2O_2S predicted by the evolutionary algorithm USPEX (See Table S1 for its structural parameters). These images are viewed along (left) *a*-axis, (middle) *b*-axis, and (right) *c*-axis. Lanthanum, Oxygen and Sulfur atoms are represented by the green, red and yellow spheres respectively. The $(La_2O_2)^{2+}$ monolayer exhibits almost tetragonal in-plane symmetry as observed in the precursor $La_2O_2S_2$. Sulfur atoms lie on the same plane without corrugation.

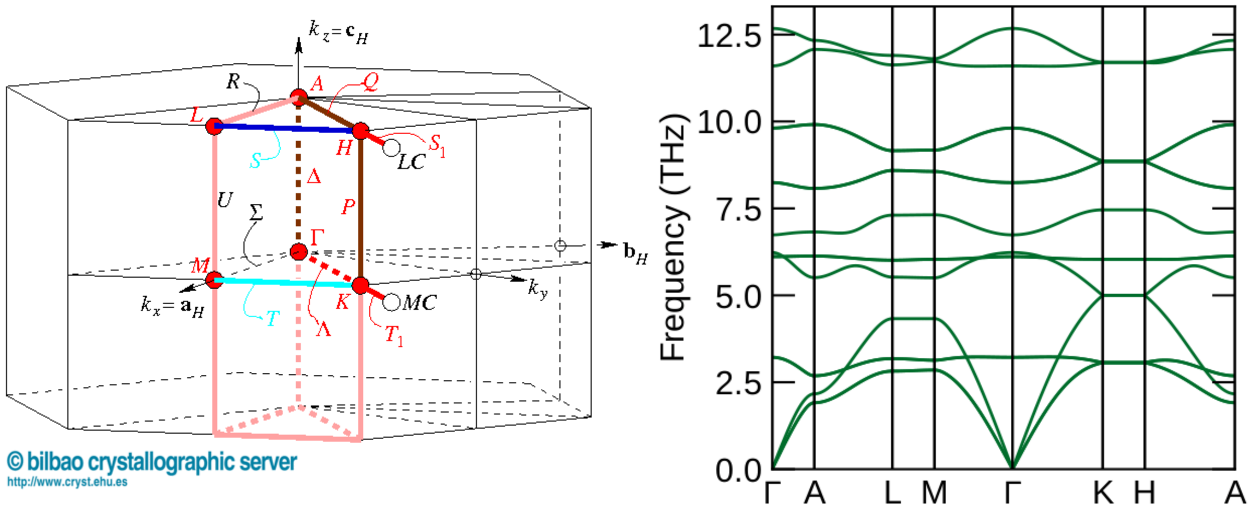

Figure S3. Brillouin zone of the predicted structure type *hP*-La_2O_2S and its phonon dispersion calculated by using DEPT at the GGA-PBE level of theory (See the experimental procedure for details).

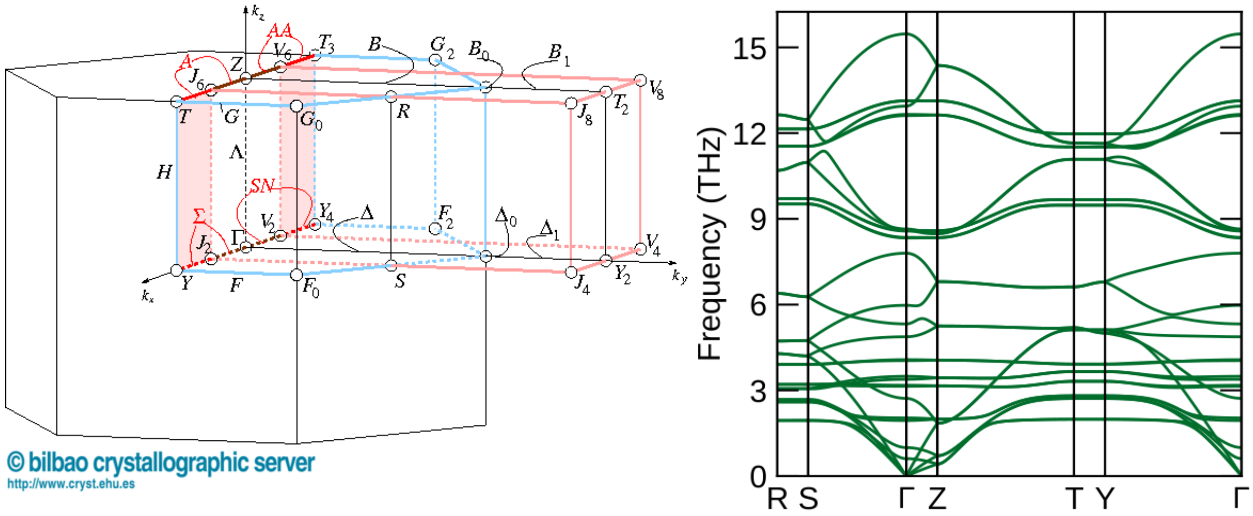

Figure S4. Brillouin zone of the predicted structure type *oA*-La_2O_2S and its phonon dispersion calculated by using DEPT at the GGA-PBE level of theory (See the experimental procedure for details).

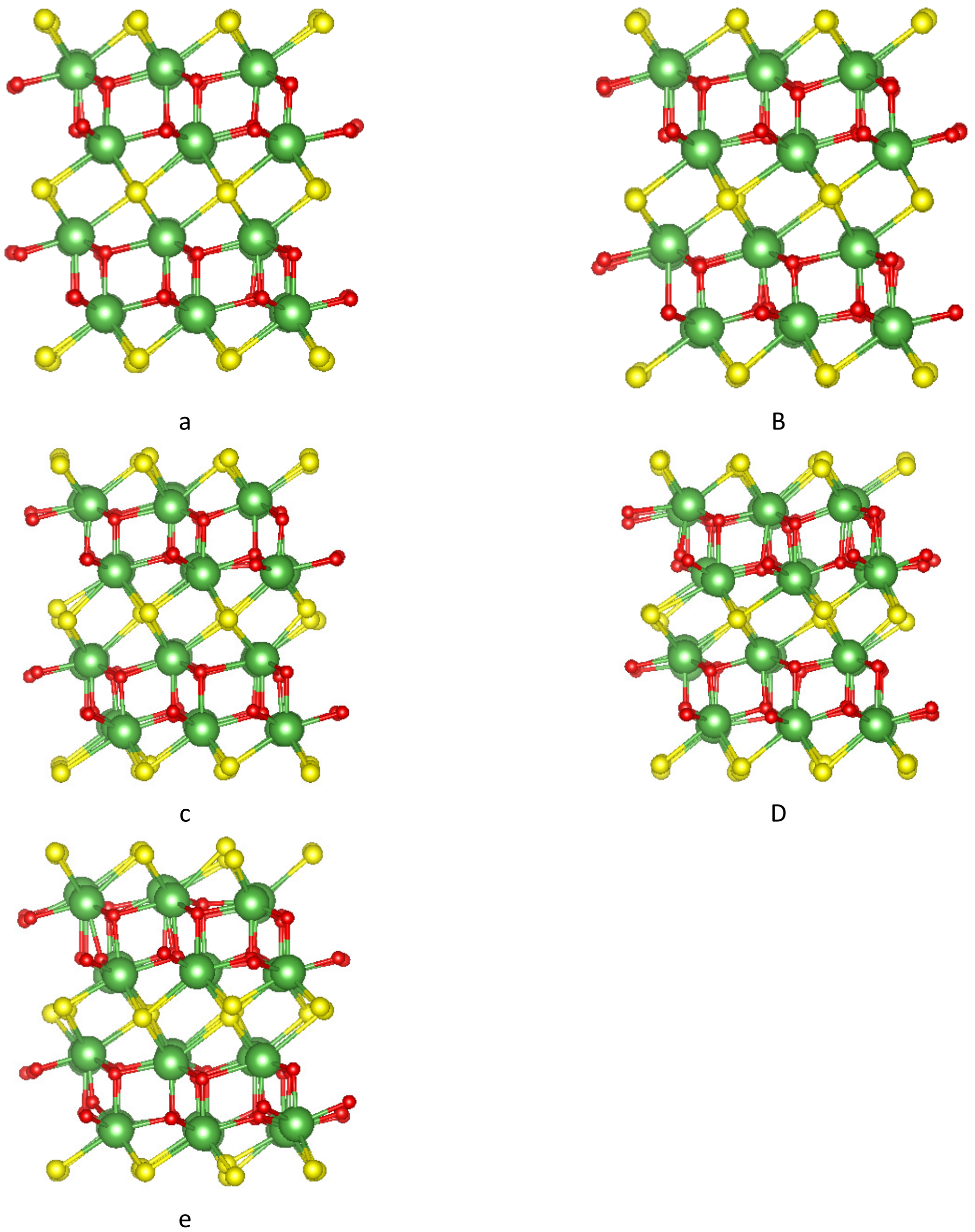

Figure S5. Snapshots of the type hP-La_2O_2S 3 × 3 × 2 supercell at the end of 10 ps AIMD simulations at (a) 300 K, (b) 600 K, (c) 900 K, (d) 1200K and (e) 1500 K. Lanthanum, Oxygen and Sulfur atoms are represented by the green, red and yellow spheres respectively. As observed on Figure S7, the hP-La_2O_2S structure is thermally stable up to 1500 K.

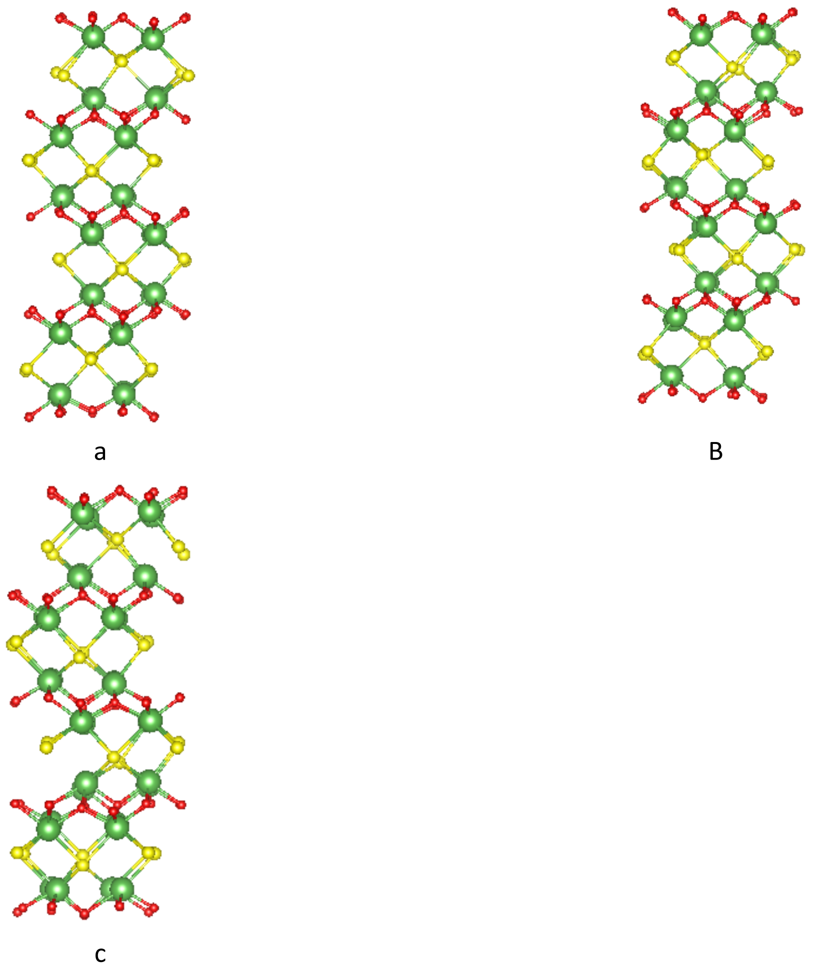

Figure S6 is shown as one image. Its three panels are labelled as follows:

Figure S6. Snapshots of the type *oA*-La_2O_2S 2 × 2 × 2 supercell at the end of 10 ps AIMD simulations at (a) 300 K, (b) 600 K and (c) 900 K. Lanthanum, oxygen and sulfur atoms are represented by the green, red and yellow spheres respectively. As observed in the figure, the *oA*-La_2O_2S structure is thermally stable up to 600 K.

Experimental results

1. Characterization of *oA*-La_2O_2S

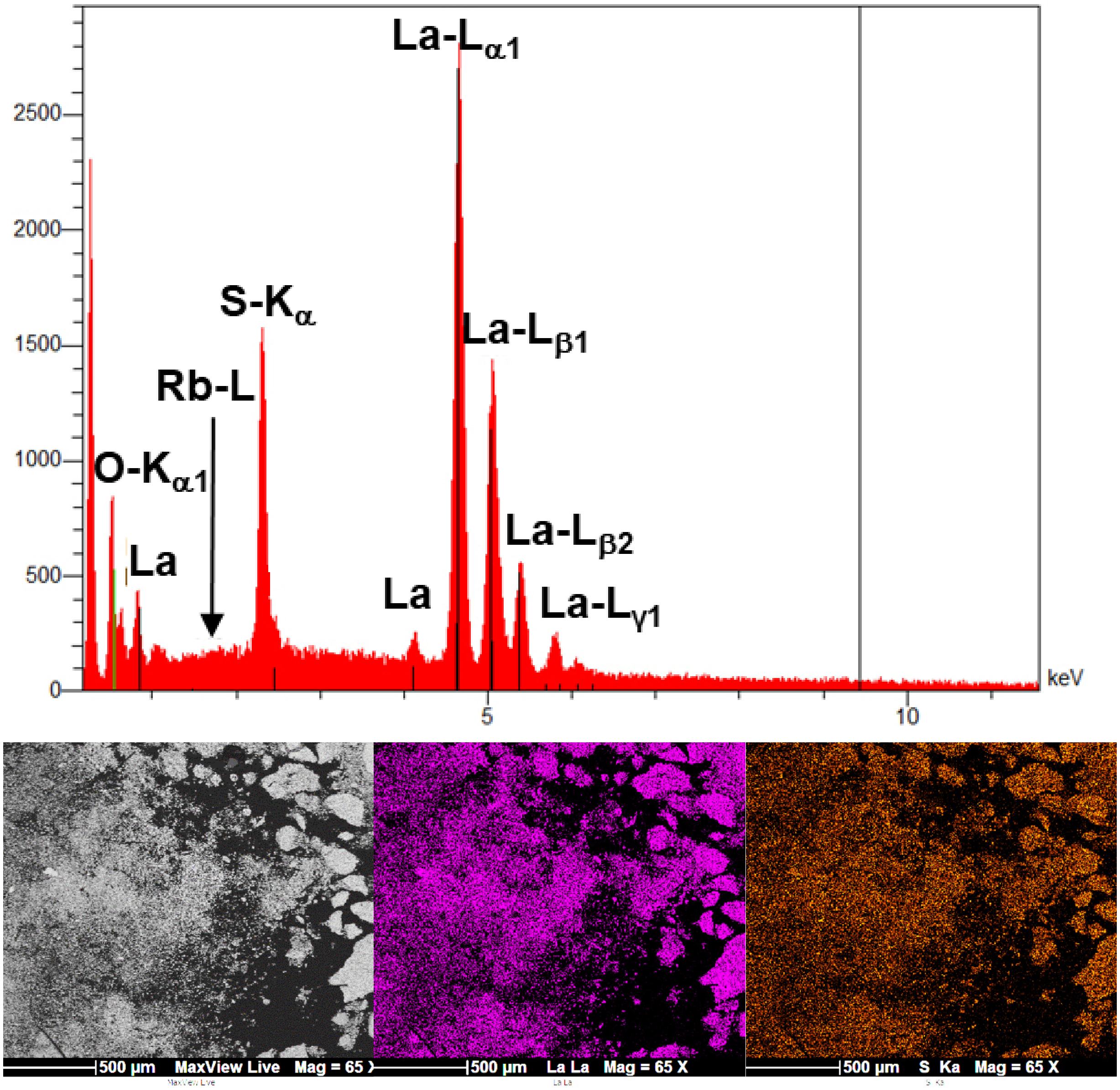

Figure S7. (a) EDX spectrum of the *oA*-La_2O_2S powder sample impregnated with the epoxy resin and (b) its backscattered electron image (BEI) as well as its elemental composition mapped for La and S.

Table S3. Crystallographic parameters determined from Rietveld refinement of *oA*-La_2O_2S powder.

Crystallographic data				
Chemical formula	La_2O_2S			
Molar mass (g mol^{-1})	341.87			
Symmetry	Orthorhombic			
Color	White			
Space group	*Amm2* (No. 38)			
a (Å)	4.1489(0)			
b (Å)	3.9750(9)			
c (Å)	12.727(9)			
Volume (Å^3)	209.9(1)			
Z	2			
Density (g cm^{-3})	5.4088			
Anisotropic strain (Å^{-2})^2	S_{400} = 11.7(9); S_{040} = 8.60(1); S_{004} = 0.0481(0); S_{220} = 2.81(9); S_{202} = 0.829(4); S_{022} = 0.634(3);			
March-Dollase parameter (Preferred orientation along <100>)	P_{md} = 0.943(1)			
Structural refinement				
Profile reliability factor	R_p = 6.39%			
Weighted profile reliability factor	R_{wp} = 8.74%			
Bragg reliability factors	*R*(obs) = *R*(all) = 1.68%			
Weighted Bragg profile reliability factors	R_w(obs) = R_w(all) = 2.29%			
Goodness of fit	χ^2 = 1.33			
Atomic positions and isotropic thermal parameters[a]				
Atom	*x*	*y*	*z*	U_{iso} (Å^2)
La1	0	0	0.6441(9)	0.0055(4)
La2	0.5	0.5	0.8379(7)	0.0040(2)
O1	0	0.5	0.7360(4)	0.005[b]
O2	0.5	0	0.7180(0)	0.005[b]
S1	0	0	0.9665(4)	0.0066(1)

[a] Site-occupancy factors of all atoms are fixed to full occupancy.
[b] These atomic displacement factors are fixed to 0.005.

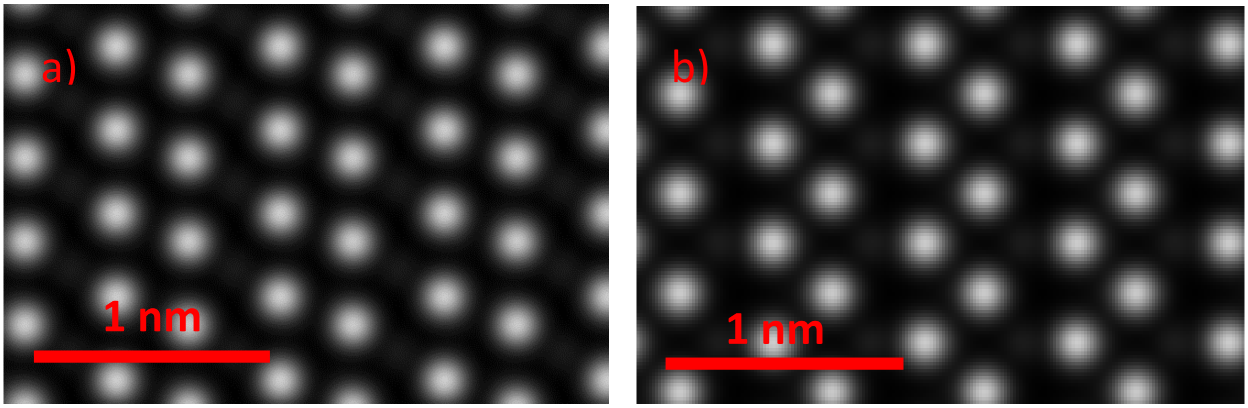

Figure S8. Comparison of simulated HAADF images with the stable phase *hP*-La_2O_2S (a) and the new *oA*-La_2O_2S (b) along [001]

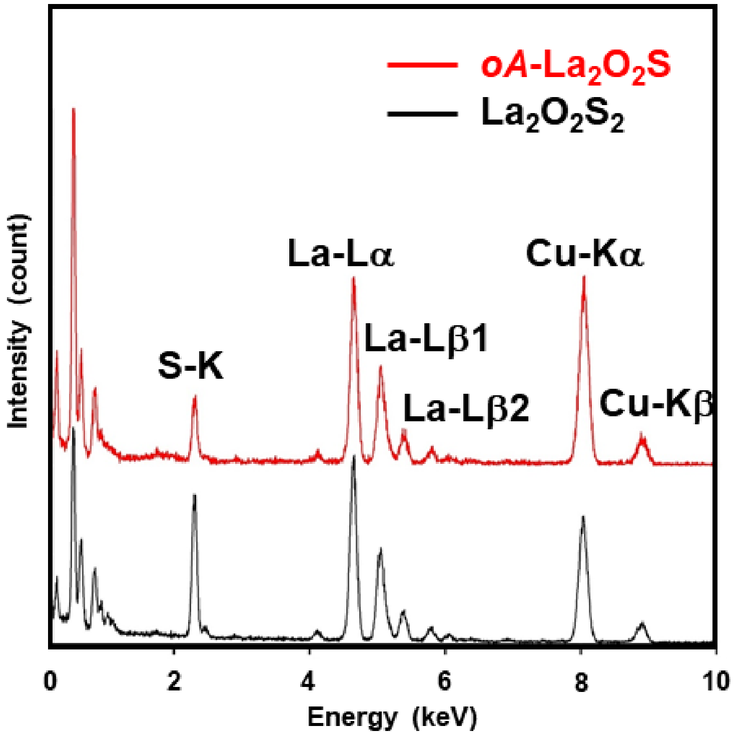

Figure S9. Energy-dispersive X-ray (EDX) spectra of *oA*-La_2O_2S (red) and $La_2O_2S_2$ (black). The measurements were carried out on their single crystals (≤ 100 nm) by STEM.

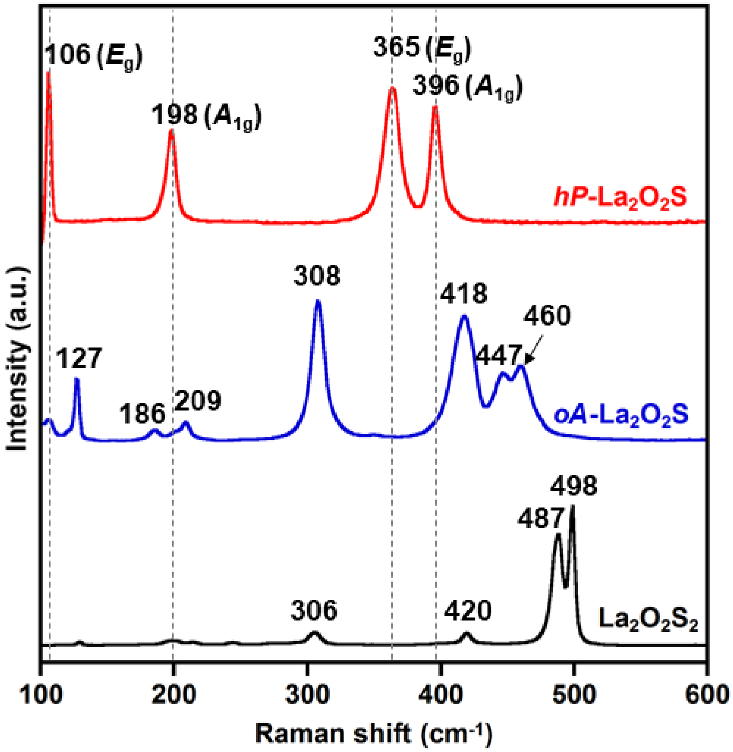

Figure S10. Raman spectra of $La_2O_2S_2$, *oA*-La_2O_2S and *hP*-La_2O_2S. *oA*-La_2O_2S was prepared by the topochemical reaction of $La_2O_2S_2$ with Rb (*vide supra*). *hP*-La_2O_2S was obtained from the thermal treatment of $La_2O_2S_2$ under 5% H_2 / Ar at 500 °C (See Figure S12). Raman spectrum of *hP*-La_2O_2S was found identical with that reported by Yokono *et al.*[9] and thus assigned in accordance with their factor group analysis. Laser power: 0.15 mW.

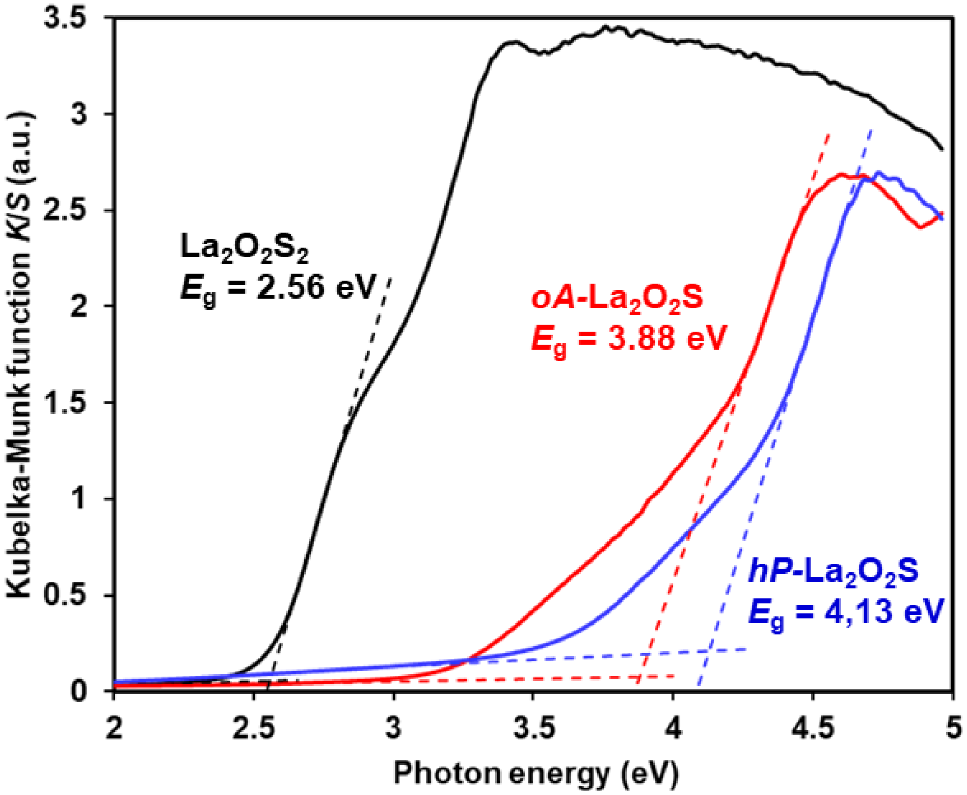

Figure S11. Diffuse-reflectance spectra of $La_2O_2S_2$, *oA*- and *hP*-La_2O_2S and their bandgap E_g. The difference in E_g values between *oA*-La_2O_2S and *hP*-La_2O_2S is related to the change of conformation of the La_2O_2 layer inducing a change of the coordination sphere of the sulfur atoms (trigonal antiprism in *hP* vs trigonal prism in *oA*). The decrease of the gap going from *oA*-La_2O_2S to $La_2O_2S_2$ is directly related to the formation of the S-S pairs in the latter compound. It leads to a change of character of the conduction band from the La(5d) band to the lower lying 3p σ* level of the S-S bond.

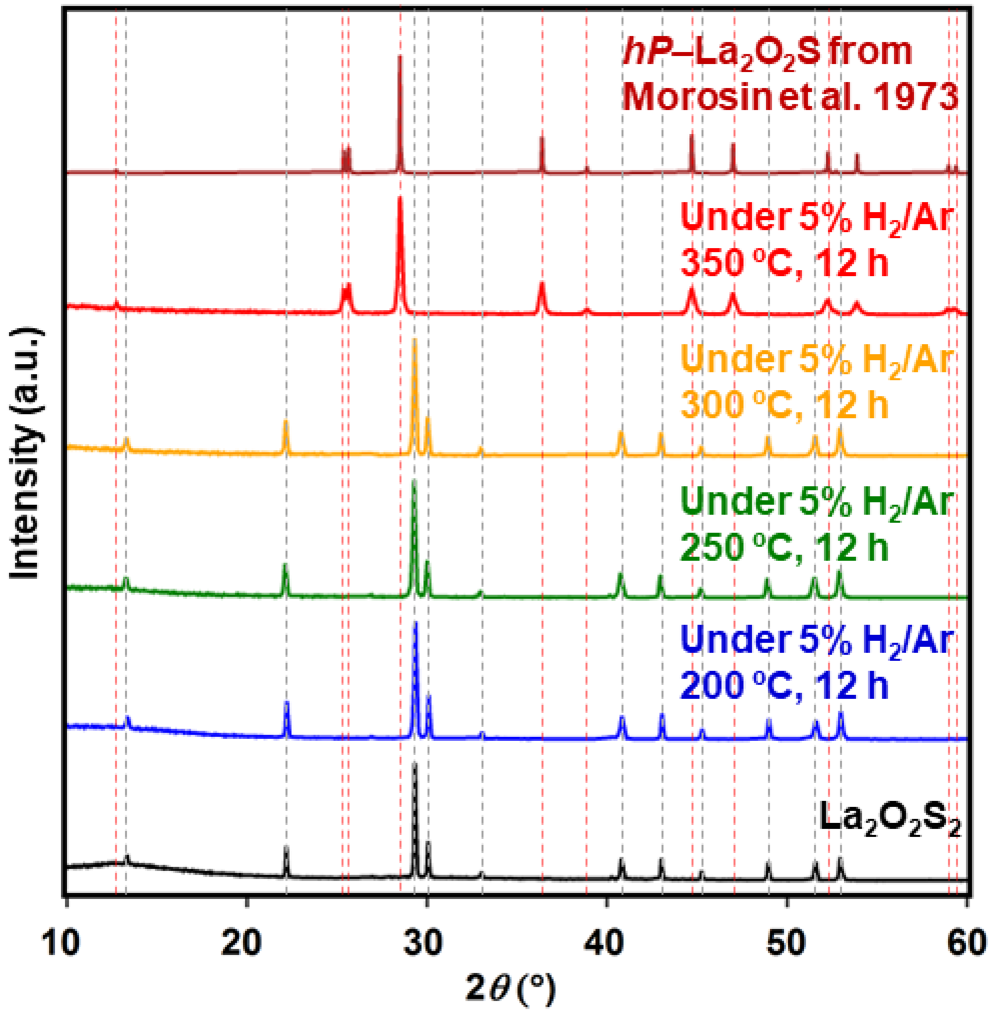

Figure S12. XRD patterns after the thermal treatment of $La_2O_2S_2$ under 5% H_2 / Ar flow at 200-350 °C. For comparison also the theoretical XRD pattern of *hP*-La_2O_2S reproduced from the literature[10] was displayed.

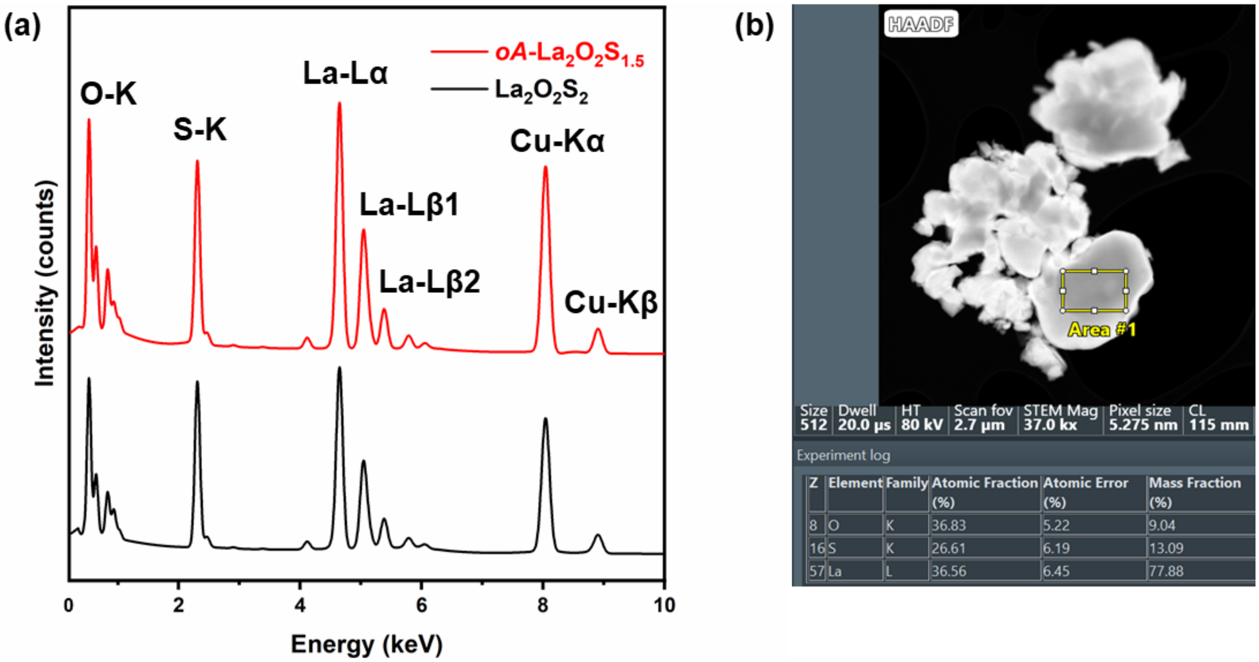

Figure S13. (a) Energy-dispersive X-ray (EDX) spectra of *oA*-$La_2O_2S_{1.5}$ (red) and $La_2O_2S_2$ (black) found from the sample after the thermal treatment of *oA*-La_2O_2S + 0.5 S at 200 °C (see Fig. 3). Each measurement was carried out on the respective grains selected on STEM (b) Image of the grain selected for *oA*-$La_2O_2S_{1.5}$, and results of the EDX analysis in the area #1 showing a La / O / S ratio of 2.0(3) / 2.0(3) / 1.5(3), supporting the expected composition $La_2O_2S_{1.5}$.

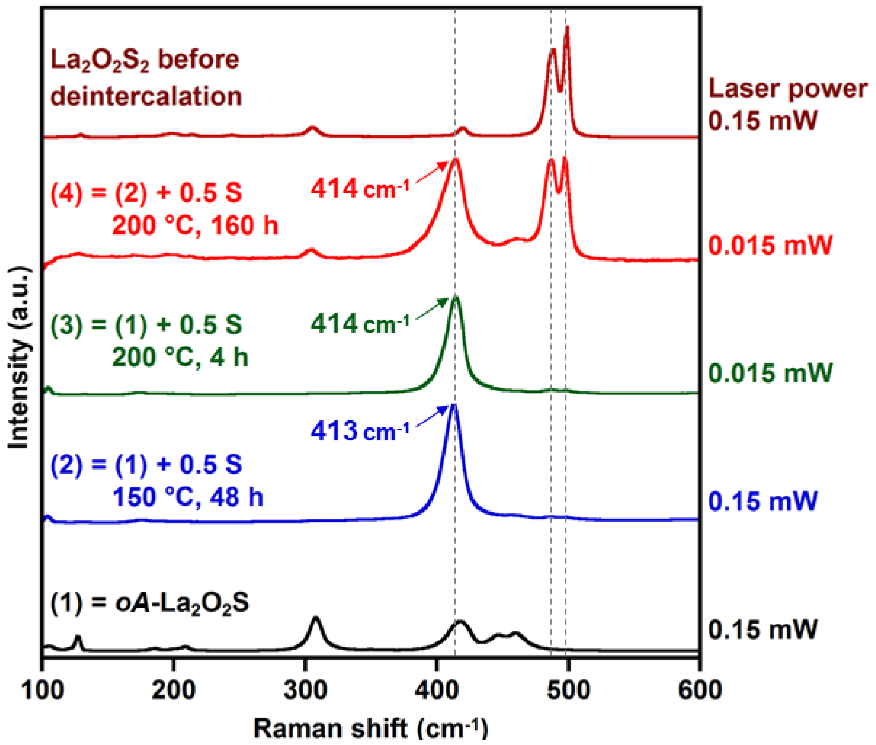

Figure S14. Raman spectra of *oA*-La_2O_2S and its mixture with sulfur after thermal treatments under 150– 200 °C. For comparison, also the Raman spectrum of the pristine $La_2O_2S_2$ is displayed.

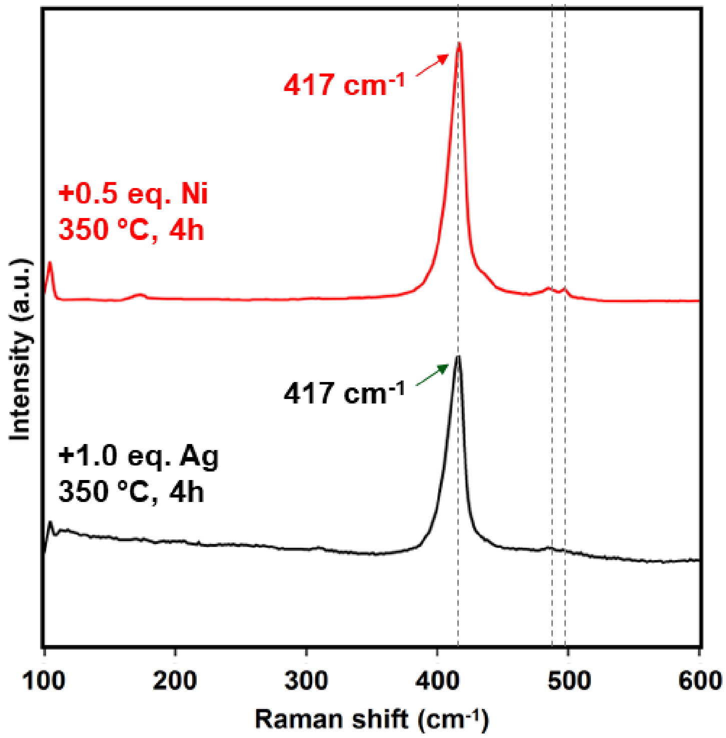

Figure S15. Raman spectra of $La_2O_2S_2$ and its mixture with Ag and Ni after the thermal treatments at 350 °C.

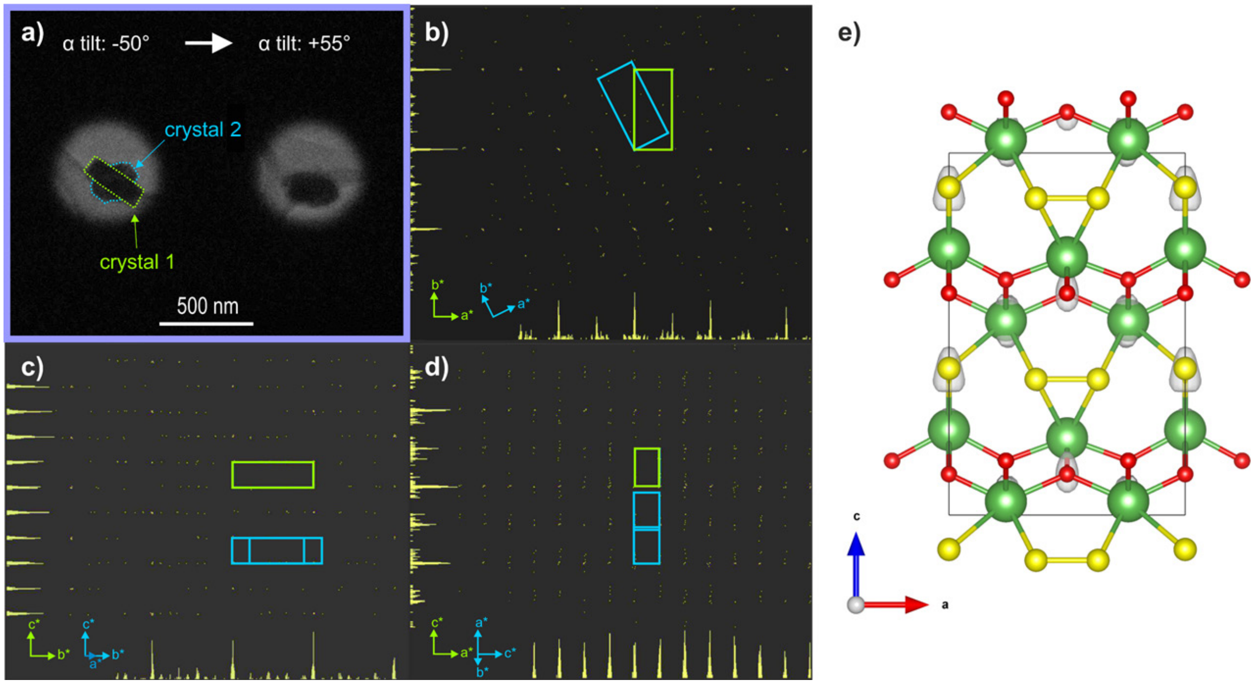

Figure S16. a) Picture of the two superimposed crystals used in the structure analysis of $La_2O_2S_{1.5}$. Projections of the reciprocal space reconstructed in PETS2.0 following b) c* (crystal 1), c) a* and d) b* showing the unit cells of the two crystals measured in the same PEDT data set. The green and blue indexing correspond to the crystal 1 and 2 respectively. e) Structure solved in SUPERFLIP and superimposed with electrostatic potential map represented as isosurfaces.

Table S4. Summary of PEDT data and its refinement.

Unit–cell parameters (PEDT)	
Refined structural formula	$La_2O_2S_{1.5}$
A	8.348 Å
B	3.961 Å
C	12.645 Å
V	418.1 Å^3
Z	4
Density [g.cm^{-3}]	5.6864
Space group	*Amm2*
Data Acquisition	
Temperature	ambient
TEM	Philips CM120
Radiation (wavelength)	electrons (0.0335Å)
Resolution range sinθ/λ	0.1–0.7 Å^{-1}
Limiting Miller indices	0< *h* < 11, 0< *k* < 5, -17 < *l* < 17
No. of independent reflections (obs/all) – kinematic	696/2905
R_{int} (obs/all) – kinematic	cryst.1: 16.33%/16.47%, cryst. 2: 20.30%/21.77%
Redundancy	4.115
Coverage for sinθ/λ = 0.7Å^{-1}	97.88 %
Kinematical refinement	
No. of reflections (obs/all)	1803/7132
R, *wR* (obs)	0.2360/0.3009
N parameters	11
Dynamical refinement	
RSg(max) (cryst.1, cryst. 2)	0.55
No. of reflections (obs/all)	all: 3523/4246<br>cryst.1.: 1836/2130<br>cryst.2.: 1687/2116
R, *wR* (obs)	all: 0.1012/0.1142<br>cryst.1.: 0.0941/0.1027<br>cryst.2.: 0.1105/0.1301
N parameters/*N* struct. parameters	200/13
Crystal thickness (cryst. 1, cryst. 2)	428(2) Å and 354(3) Å

Atomic positions and isotropic thermal parameters

Atom	*wyck.*	*x/a*	*y/b*	*z/c*	*Uiso* [Å^2]
La1	4c	0.2647(2)	0.5	0.1144(6)	0.0099(3)
La2	2b	0.5	0.5	0.4182(7)	0.0099(3)
La3	2a	0	0.5	0.4387(7)	0.0099(3)
O1	2a	0	0.5	0.0440(12)	0.0017(8)
O2	2b	0.5	0.5	0.0103(11)	0.0017(8)
S1	4c	0.1204(7)	0	0.2768(8)	0.0114(9)
S2	2b	0.5	0	0.2367	0.0114(9)
O3	4c	-0.2542(9)	0.5	0.5123(10)	0.0017(8)

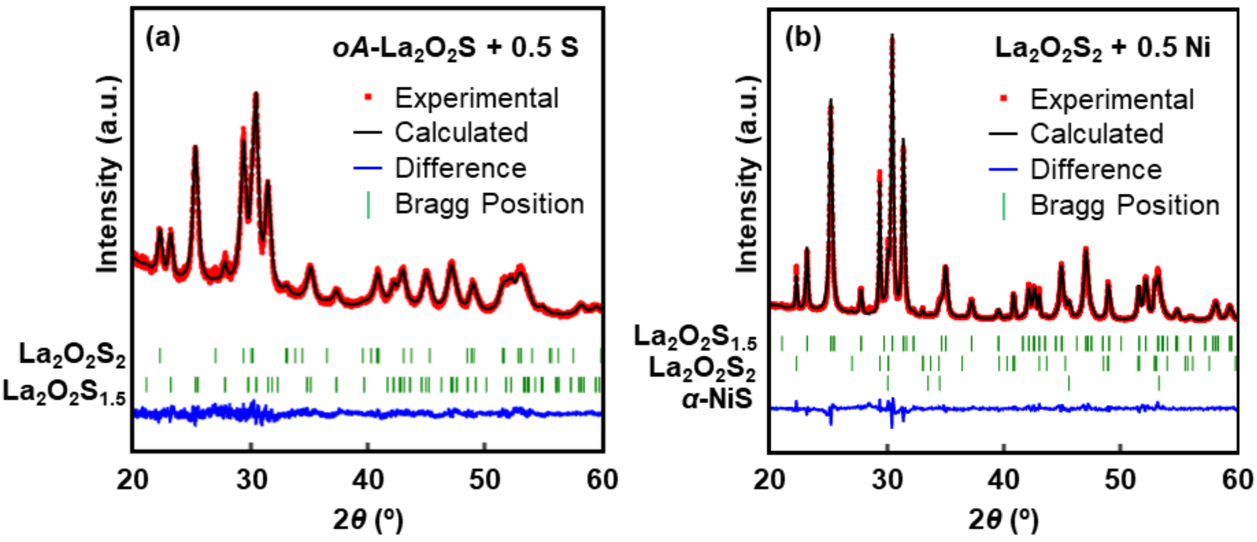

Figure S17. Rietveld refinements of the XRD patterns obtained from (a) the reaction mixture *oA*-La_2O_2S + 0.5 S after the thermal treatment under 200 °C for 4h and (b) the reaction mixture $La_2O_2S_2$ + 0.5 Ni after the thermal treatment under 350 °C for 4 h.

Table S5. Crystallographic parameters determined from Rietveld refinement of the mixture *oA*-La_2O_2S + 0.5 S after the thermal treatments at 200 °C for 4 h.

Basic data	
Color	Pale yellow
Structural refinement – Profile	
Profile reliability factor	R_p = 3.60%
Weighted profile reliability factor	R_{wp} = 4.98%
Crystallographic data – $La_2O_2S_{1.5}$	
Chemical formula	$La_2O_2S_{1.5}$
Molar mass (g mol^{-1})	357.90
Symmetry	Orthorhombic
Space group	*Amm2* (No. 38)
a (Å)	8.4329(4)
b (Å)	4.0272(5)
c (Å)	12.832(1)
Volume (Å^3)	435.8(0)
Z	4
Density (g cm^{-3})	5.4549
Anisotropic strain (Å^{-2})^2	S_{400} = 4.05(9); S_{040} = 38.8(9); S_{004} = 0.405(5); S_{220} = 5.96(1); S_{202} = 0.869(0); S_{022} = 1.669(4);
Mass fraction	0.656

Structural refinement – $La_2O_2S_{1.5}$	
Bragg reliability factors	R(obs) = R(all) = 1.26%
Weighted Bragg profile reliability factors	R_w(obs) = R_w(all) = 1.43%

Atomic positions and isotropic thermal parameters – $La_2O_2S_{1.5}$ [a]

Atom	x	y	z	U_{iso} (Å^2)
La1	0.2648(3)	0.5	0.1161(7)	0.0099(3)
La2	0.5	0.5	0.4255(4)	0.0099(3)
La3	0	0.5	0.4324(8)	0.0099(3)
O1	0	0.5	0.0925(5)	0.0017(8)
O2	0.5	0.5	0.0275(25)	0.0017(8)
O3	-0.2674(0)	0.5	0.4941(1)	0.0017(8)
S1	0.0925(75)	0	0.2623(4)	0.0114(9)
S2	0.5	0	0.2272(9)	0.0114(9)

Crystallographic data – $La_2O_2S_2$ [b]	
Chemical formula	$La_2O_2S_2$
Molar mass (g mol^{-1})	373.93
Symmetry	Orthorhombic
Space group	*Cmca* (No. 64)
a (Å)	13.217(0)
b (Å)	5.9210(8)
c (Å)	5.9483(7)
Volume (Å^3)	465.5(1)
Z	4
Density (g cm^{-3})	5.3354
Anisotropic strain (Å^{-2})^2	S_{400} = 0.595(8); S_{040} = 1.05(0); S_{004} = -1.07(6); S_{220} = 1.72(0); S_{202} = 0.986(5); S_{022} = 3.054(0);
Mass fraction	0.344
Structural refinement – $La_2O_2S_2$	
Bragg reliability factors	R(obs) = R(all) = 1.63%
Weighted Bragg profile reliability factors	R_w(obs) = R_w(all) = 1.69%

[a] Site-occupancy factors of all atoms were fixed to full occupancy. Isotropic thermal parameters were fixed to the same values as obtained by the PEDT analysis (See Table S4).

[b] The $La_2O_2S_2$ phase was refined using the crystallographic data reported by Ostoréro et al.[11] Its atomic parameters were not refined and fixed to the same values with the reported ones.

Table S6. Crystallographic parameters determined from Rietveld refinement of the mixture $La_2O_2S_2$ + 0.5 Ni after the thermal treatments at 350 °C for 4 h.

Basic data	
Color	Black
Structural refinement – Profile	
Profile reliability factor	R_p = 4.45%
Weighted profile reliability factor	R_{wp} = 6.17%
Crystallographic data – $La_2O_2S_{1.5}$	
Chemical formula	$La_2O_2S_{1.5}$
Molar mass (g mol^{-1})	357.90
Symmetry	Orthorhombic
Space group	*Amm2* (No. 38)
a (Å)	8.4759(5)
b (Å)	4.0307(1)
c (Å)	12.853(0)
Volume (Å^3)	439.1(1)
Z	4
Density (g cm^{-3})	5.4137
Anisotropic strain (Å^{-2})^2	S_{400} = 0.590(1); S_{040} = 8.64(2); S_{004} = 0.0731(5); S_{220} = 0.752(3); S_{202} = 0.265(7); S_{022} = 0.342(1);
Mass fraction	0.772
Structural refinement – $La_2O_2S_{1.5}$	
Bragg reliability factors	*R*(obs) = *R*(all) = 1.90%
Weighted Bragg profile reliability factors	R_w(obs) = R_w(all) = 2.58%

Atomic positions and isotropic thermal parameters – $La_2O_2S_{1.5}$ [a]

Atom	*x*	*y*	*z*	U_{iso} (Å^2)
La1	0.2640(5)	0.5	0.1145(9)	0.0099(3)
La2	0.5	0.5	0.4214(1)	0.0099(3)
La3	0	0.5	0.4377(3)	0.0099(3)
O1	0	0.5	0.0201(4)	0.0017(8)
O2	0.5	0.5	0.0133(5)	0.0017(8)
O3	-0.2731(7)	0.5	0.5000(8)	0.0017(8)
S1	0.1356(5)	0	0.2625(5)	0.0114(9)
S2	0.5	0	0.2322(8)	0.0114(9)

Crystallographic data – $La_2O_2S_2$ [b]	
Chemical formula	$La_2O_2S_2$
Molar mass (g mol^{-1})	373.93
Symmetry	Orthorhombic
Space group	*Cmca* (No. 64)
a (Å)	13.219(1)
b (Å)	5.9416(8)
c (Å)	5.9344(6)
Volume (Å^3)	466.1(1)
Z	4
Density (g cm^{-3})	5.3285
Mass fraction	0.122
Structural refinement – $La_2O_2S_2$	
Bragg reliability factors	R(obs) = R(all) = 3.24%
Weighted Bragg profile reliability factors	R_w(obs) = R_w(all) = 4.38%
Crystallographic data – α-NiS [c]	
Chemical formula	NiS
Molar mass (g mol^{-1})	90.75
Symmetry	Hexagonal
Space group	$P6_3/mmc$ (No. 194)
a (Å)	3.4332(3)
c (Å)	5.3467(3)
Volume (Å^3)	54.57(9)
Z	2
Density (g cm^{-3})	5.5220
Mass fraction	0.106
Structural refinement – α-NiS	
Bragg reliability factors	R(obs) = R(all) = 2.20%
Weighted Bragg profile reliability factors	R_w(obs) = R_w(all) = 2.77%

[a] Site-occupancy factors of all atoms were fixed to full occupancy. Isotropic thermal parameters were fixed to the same values as obtained by the PEDT analysis (See Table S4).

[b] The $La_2O_2S_2$ phase was refined using the crystallographic data reported by Ostoréro et al.[11] Its atomic parameters were not refined and fixed to the same values with the reported ones.

[c] The *α*-NiS phase was refined using the crystallographic data reported by Trahan et al.[12] Its atomic parameters were not refined and fixed to the same values with the reported ones.

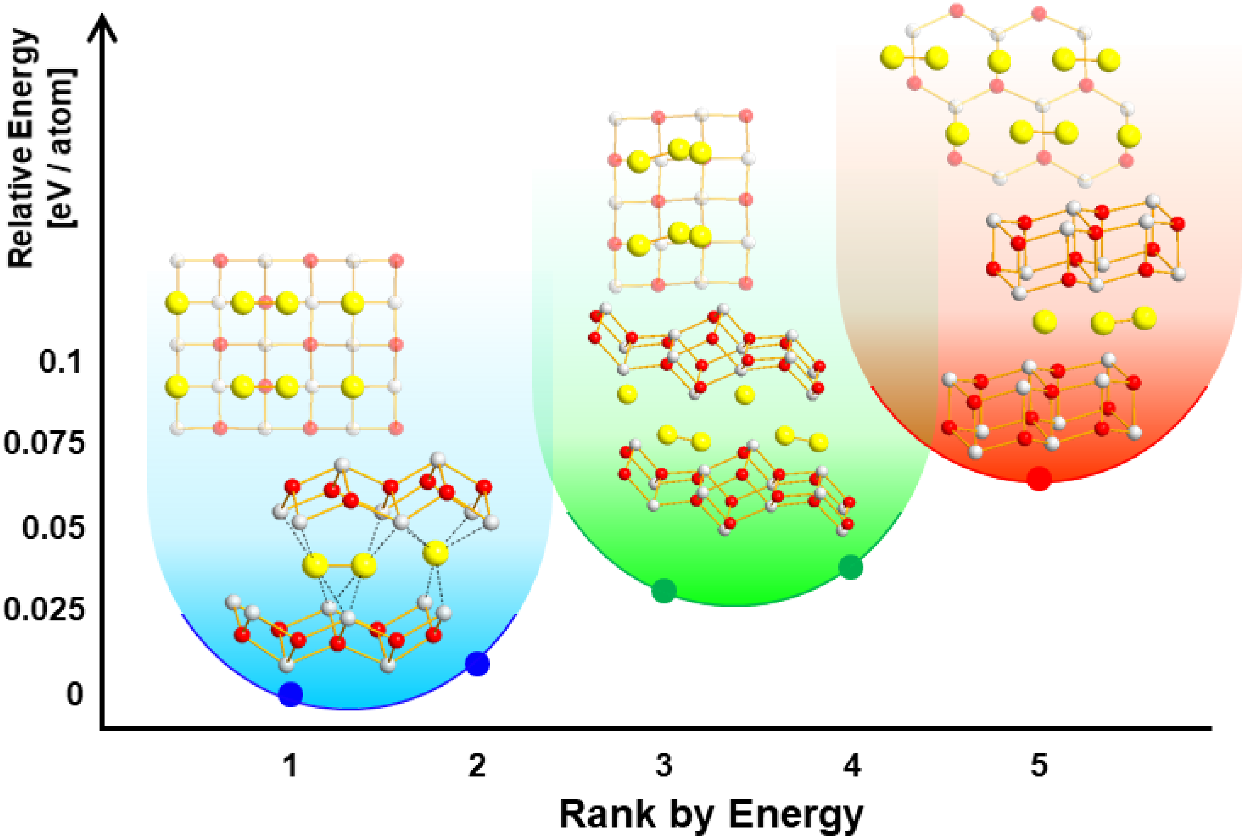

Figure S18. The most stable structures predicted for $La_2O_2S_{1.5}$ composition by USPEX calculation. Those with the same structure type (and space groups) was grouped by each colour and the more stable one in each group was displayed. Each structure type was compared with respect to its enthalpy [eV/atom] relative to the most stable (1st in the rank) structure. Compared to the most stable structure type (1st in the rank), the second and third most stable structure types (3rd and 5th in the rank, respectively) are higher in energy by 31 and 65 meV/atom, respectively.

References

1. Kortüm, G., Braun, W. & Herzog, G. Principles and techniques of diffuse - reflectance spectroscopy. *Angew. Chem. Int. Ed.* **2**, 333-304 (1963).
2. Tauc, J., Grigorovici, R. & Vancu, A. Optical properties and electronic structure of amorphous germanium. *Phys. Status Solidi* **15**, 627-637 (1966).
3. Davis, E. A. & Mott, N. F. Conduction in non-crystalline systems V. Conductivity, optical absorption and photoconductivity in amorphous semiconductors. *Phil. Mag. A* **22**, 903-922 (1970).

4. Sabatini, R., Gorni, T. & de Gironcoli, S. Nonlocal van der Waals density functional made simple and efficient. *Phys. Rev. B*, **87**, 041108 (2013).
5. Peng, H., Yang, Z.-H., Perdew, J. P. & Sun, J. Versatile van der Waals Density Functional Based on a Meta-Generalized Gradient Approximation. *Phys. Rev. X*, **6**, 041005 (2016).
6. Sun, J., Ruzsinszky, A. & Perdew, J. P. Strongly Constrained and Appropriately Normed Semilocal Density Functional. *Phys. Rev. Lett.* **115**, 036402 (2015).
7. Togo, A., Oba, F. & Tanaka, I. First-principles calculations of the ferroelastic transition between rutile-type and $CaCl_2$-type SiO_2 at high pressures. *Phys. Rev. B* **78**, 134106-134114 (2008).
8. Tasci, E. S., de la Flor, G., Orobengoa, D., Capillas, C., Perez-Mato, J. M. & Aroyo, M. I. An introduction to the tools hosted in the Bilbao Crystallographic Server, *EPJ Web of Conferences* **22**, 00009 (2012).
9. Yokono, S., Imanaga, S. & Hoshina, T. Raman Spectra for Eu Doped Ln_2O_2S Phosphors. *J. Phys. Soc. Jpn.* **46**, 1882-1888 (1979).
10. Morosin, B. & Newman, D. J. La_2O_2S structure refinement and crystal field. *Acta Cryst.* **B29**, 2647-2648 (1973).
11. Ostoréro, J. & Leblanc, M. Room-temperature structure of $La_2O_2S_2$. *Acta Cryst.* **C46**, 1376-1378 (1990).
12. Trahan, J., Goodrich, R. G. & Watkins, S. F. X-ray diffraction measurements on metallic and semiconducting hexagonal NiS. *Phys. Rev. B* **2**, 2859-2863 (1970).
